# Functional and Comparative Analysis of Centromeres Reveals Clade-Specific Genome Rearrangements in *Candida auris* and a Chromosome Number Change in Related Species

**DOI:** 10.1128/mBio.00905-21

**Published:** 2021-05-11

**Authors:** Aswathy Narayanan, Rakesh Netha Vadnala, Promit Ganguly, Pavitra Selvakumar, Shivaprakash M. Rudramurthy, Rajendra Prasad, Arunaloke Chakrabarti, Rahul Siddharthan, Kaustuv Sanyal

**Affiliations:** a Molecular Mycology Laboratory, Molecular Biology and Genetics Unit, Jawaharlal Nehru Centre for Advanced Scientific Research, Bangalore, India; b Computational Biology, The Institute of Mathematical Sciences/HBNI, Chennai, India; c Department of Medical Microbiology, Postgraduate Institute of Medical Education and Research, Chandigarh, India; d Amity Institute of Biotechnology, Amity University Haryana, Haryana, India; e Osaka University, Suita, Japan; University of Texas Health Science Center

**Keywords:** *Candida haemulonii*, fungal pathogen, centromere inactivation, geographical clades, karyotype evolution

## Abstract

The thermotolerant multidrug-resistant ascomycete Candida auris rapidly emerged since 2009 causing systemic infections worldwide and simultaneously evolved in different geographical zones. The molecular events that orchestrated this sudden emergence of the killer fungus remain mostly elusive. Here, we identify centromeres in C. auris and related species, using a combined approach of chromatin immunoprecipitation and comparative genomic analyses. We find that C. auris and multiple other species in the *Clavispora/Candida* clade shared a conserved small regional GC-poor centromere landscape lacking pericentromeres or repeats. Further, a centromere inactivation event led to karyotypic alterations in this species complex. Interspecies genome analysis identified several structural chromosomal changes around centromeres. In addition, centromeres are found to be rapidly evolving loci among the different geographical clades of the same species of C. auris. Finally, we reveal an evolutionary trajectory of the unique karyotype associated with clade 2 that consists of the drug-susceptible isolates of C. auris.

## INTRODUCTION

First isolated from an infected ear of a patient in Japan in 2009, Candida auris emerged as a multidrug-resistant opportunistic fungal pathogen causing nosocomial infections worldwide in a short time span (1–5). It can survive at elevated temperatures and high salt concentrations, which otherwise act as physiological barriers to fungal infections (6, 7). As a haploid ascomycete, C. auris often displays exceptional resistance to major antifungals like azoles and common sterilization agents, rendering it a difficult pathogen to treat (8–10). As an opportunistic pathogen, C. auris colonizes skin and causes systemic infections, thereby posing threats to patients with other clinical conditions like diabetes mellitus, chronic renal disease, and, more recently, COVID-19 infections (11, 12). C. auris emerged and evolved simultaneously as distinct geographical clades—South Asian (clade 1), East Asian (clade 2), South African (clade 3), South American (clade 4), and a potential fifth clade from Iran (13, 14). The clades are separated by tens of thousands of single nucleotide polymorphisms but exhibit clonality within a clade (13). The mechanisms that underlie the sudden emergence and spread of C. auris as distinct geographical clades, though mostly unknown, represent rapid evolution modes in a fungal pathogen.

A pathogen evolves in nature to survive the evolutionary arms race. Genetic diversity is a prerequisite for the pathogen to adapt to changing conditions. In the absence of sexual reproduction, chromosomal reshuffling serves to generate diversity in some predominantly asexual fungal pathogens (15–18), thereby circumventing evolutionary dead ends. Chromosomal rearrangements and aneuploidy are also known to enhance drug resistance and virulence in primarily asexual fungi (19–21). Centromeres (*CEN*s), which appear as the primary constrictions on metaphase chromosomes, are emerging as a central hub of such chromosomal rearrangements contributing to karyotype diversity and speciation (22). Centromeres exhibit diversity in their properties like the length of centromeric chromatin, repeat/transposon content, and GC-richness. However, centromeric chromatin in most species is occupied by the *CEN*-specific histone variant CENP-A^Cse4^, which replaces canonical histone H3 in the centromeric nucleosomes and is regarded as the epigenetic hallmark defining *CEN* identity (23, 24). Centromeric chromatin also provides the foundation for assembling several multiprotein complexes to form the kinetochore. Dynamic interactions of spindle microtubules and kinetochores result in the precise segregation of sister chromatids in daughter cells during cell division. Centromere clustering near the nuclear periphery is a conserved feature across the fungal kingdom (25–28). Due to spatial proximity, centromeres with homologous DNA sequences often participate in chromosomal rearrangements that result in chromosomal shuffling which can drive karyotype evolution and chromosome number alterations, contributing to the emergence of a new species (16, 17, 29).

C. auris is a sister species of three multidrug-resistant pathogens, namely, Candida haemulonii, Candida duobushaemulonii, and Candida pseudohaemulonii. These species are also closely related to another human fungal pathogen, Candida lusitaniae, and together are classified under the C*lavispora/Candida* clade of the family Metschnikowiaceae (Order: Saccharomycetales) (30, 31). Centromeres are susceptible to breaks in other fungal pathogens (16, 17, 32) and are likely to contribute to the vast karyotype diversity exhibited by C. auris (33). We believed that studying the centromere structure and function in the *C. haemulonii* species complex and associated species may reveal mechanisms/events underlying the rapid evolution of the multidrug-resistant fungal pathogen C. auris. In this study, we identified centromeres in all four clades of C. auris and leveraged the information to locate centromeres in the *C. haemulonii* complex species. Functional identification of centromeres combined with comparative genome analysis in these group of species helped us propose that a centromere inactivation event from an ancestral species facilitated genome innovations that contributed to the clade-specific parallel evolution of C. auris.

## RESULTS

### *C. auris* possesses small regional CENP-A^Cse4^-rich, GC-poor, repeat-free centromeres.

**FIG 1:**
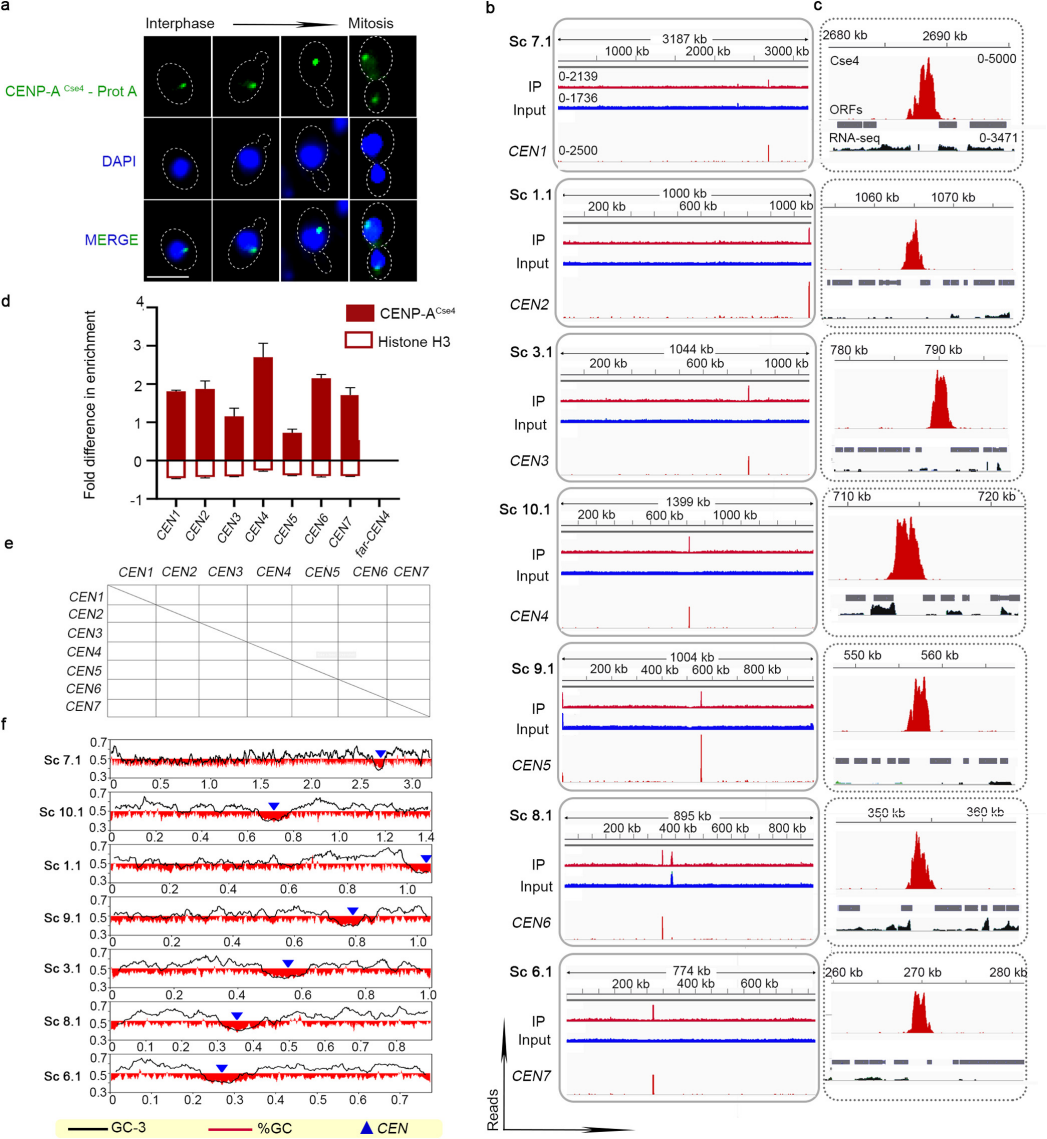
CENP-A^Cse4^-rich unique DNA sequences that are significantly depleted of histone H3 define small regional centromeres (*CEN*s) in each of seven chromosomes in C. auris clade 1. (a) Indirect immunolocalization of protein A-tagged CENP-A^Cse4^ (green) shows centromeres are clustered at the periphery of the nucleus stained with DAPI (blue) at various stages of the cell cycle. Scale bar, 3 μm. (b) CENP-A^Cse4^ ChIP-seq reads. Input, total DNA; IP, immunoprecipitated DNA; *CEN*, input subtracted from IP. (c) Zoomed-in CENP-A^Cse4^ ChIP-seq peaks (red) along with ORFs (gray) and mapped RNA-seq reads (black). The peak values are indicated. (d) Fold difference in CENP-A^Cse4^ and histone H3 enrichment at the *CEN*s compared to a control region (*far-CEN4*). qPCR values from three technical replicates are shown. The experiments were repeated three times. Error bars indicate the standard errors of the mean (SEM). Statistical analysis: one-way ANOVA; ****, *P* < 0.0001. (e) Dot plot analysis revealed the absence of repeats and the unique nature of *CEN* DNA sequences. (f) *CEN* positions (blue triangles) overlap with GC (red) and GC3 (black) scaffold minima. Coordinates (in Mb) are shown on the *x* axis, and the %GC is shown on the *y* axis. The red bars show the %GC by depicting the amount of deviation from 50% GC (above midline if values are >50% and below if values are <50%).

The histone H3 variant CENP-A^Cse4^ is exclusively associated with centromeric nucleosomes. The homolog of CENP-A^Cse4^ was identified in C. auris, using the C. albicans CENP-A^Cse4^ protein sequence as the query against the C. auris genome (GenBank assembly GCA_002759435.2 of the clade 1 isolate B8441) (30). The putative C. auris CENP-A^Cse4^ protein is 136 amino acids long and shares a 72% sequence identity with the C. albicans homolog (C3_00860W_A) (see Fig. S1 in the supplemental material). Previous studies suggested that the haploid genome of C. auris is distributed in seven chromosomes (30). To locate centromeres on each chromosome, we constructed a strain CauI46 expressing protein A-tagged CENP-A^Cse4^ from a clade 1 Indian isolate Cau46 (see Fig. S2a). Immunofluorescence staining using anti-protein A antibodies revealed punctate localization of CENP-A^Cse4^ at the nuclear periphery, suggesting typical kinetochore clustering at interphase and mitotic stages of the cell cycle (Fig. 1a). High amino acid sequence similarities with other proteins of the CENP-A family and typical localization patterns of the clustered centromeres at the nuclear periphery confirmed that the identified protein is, indeed, CENP-A^Cse4^ in C. auris. To identify CENP-A^Cse4^ associated DNA sequences as centromeric chromatin on each chromosome of C. auris, we performed CENP-A chromatin immunoprecipitation (ChIP), followed by sequencing (ChIP-seq), in strain CauI46. Sonicated genomic DNA without antibodies was also subjected to high-throughput sequencing that served as the input DNA control. The CENP-A^Cse4^ ChIP-seq analysis identified a single-peak in each of the 7 different scaffolds of 15 scaffolds of the publicly available, fragmented genome assembly of the clade 1 isolate B8441 (Fig. 1b) (30). The CENP-A^Cse4^ enriched centromeric chromatin across chromosomes spans 2,516 to 2,908 bp, with an average length of 2,727 bp (Table 1). Further analysis of these regions suggests that CENP-A^Cse4^-enriched core centromere (*CEN*) loci in C. auris are largely devoid of open reading frames (ORFs) and represent poly(A) transcriptional cold spots (Fig. 1c). To further confirm ChIP-seq results, ChIP-quantitative PCR (ChIP-qPCR) using specific primers was performed to measure CENP-A^Cse4^ abundance at *CEN*s compared to a noncentromeric genomic locus, ∼200 kb away from *CEN4* (*far-CEN4*). The same centromeric and noncentromeric primer pairs (see Table S3) were used to assess the canonical histone H3 occupancy in the corresponding regions by histone H3 ChIP-qPCR analysis. As expected, histone H3 levels were significantly depleted at the *CEN*s compared to the *far-CEN* region (Fig. 1d). Binding of CENP-A^Cse4^ to transcriptionally inert, histone H3-depleted loci of comparable length on different contigs strongly indicates that these genomic regions correspond to authentic centromeric chromatin.

**TABLE 1:** Centromere features in clade 1 isolate of C. auris

CEN	Scaffold no. in reference assembly (strain B8441)	Coordinates		Length (bp)	
		Start	End	CENP-A^Cse4^-enriched region	ORF-free region
*CEN1*	PEKT02000007.1	2686849	2689484	2,635	2,576
*CEN2*	PEKT02000001.1	1063461	1066333	2,872	2,398
*CEN3*	PEKT02000003.1	788992	791542	2,550	2,244
*CEN4*	PEKT02000010.1	712902	715418	2,516	2,081
*CEN5*	PEKT02000009.1	555667	558575	2,908	2,396
*CEN6*	PEKT02000008.1	352635	355378	2,743	2,192
*CEN7*	PEKT02000006.1	268329	271195	2,866	2,141

10.1128/mBio.00905-21.1 FIG S1 Degree of sequence conservation proving the identity of CENP-A^Cse4^ in C. auris and related species. Amino acid sequences of CENP-A^Cse4^ from different ascomycetes were aligned using BioEdit (v7.2) (1). *Scer*, S. cerevisiae; *Calb*, C. albicans; *Cdub*, C. dubliniensis; *Clus*, C. lusitaniae; *Cau*, C. auris; *Chae*, *C. haemulonii*; *C. duo*, *C. duobushaemulonii*; *Cphae*, *C. pseudohaemulonii*; *Cfruc*, *C. fructus*. The diverging N-terminal tail is highlighted in gray, and the conserved histone fold domain is highlighted in green. The conserved structures within the histone fold domain are also shown. Download FIG S1, TIF file, 2.4 MB. Copyright © 2021 Narayanan et al. 2021 Narayanan et al. https://creativecommons.org/licenses/by/4.0/ This content is distributed under the terms of the Creative Commons Attribution 4.0 International license.

10.1128/mBio.00905-21.2 FIG S2 The AT-rich centromeres in C. auris lack pericentromeric heterochromatin. (a) Design of tagging construct with the TAP tag and nourseothricin *N*-acetyltransferase (NAT) selection marker; western blot showing the expression of tagged CENP-A^Cse4^. The size of the corresponding band in the ladder (middle lane) is marked. (b) Dot plot comparing *CEN1* against itself identified the poly(A) stretch (zoomed-in view, blue box). Motif search identified the poly(A) stretch in all the centromere sequences (output logo from MEME suite, in red). (c) Schematic comparing the lengths of the CENP-A^Cse4^-enriched region and ORF-free region at the centromeres in C. auris (drawn to scale, shown on the *x* axis in kb). Box plots comparing the expression levels of 10 *CEN*-flanking genes (d) and *CEN*-overlapping genes (e) with the global gene expression level. The middle yellow line shows the median value. The box represents the 25th percentile (Q1) to 75th percentile (Q3) values. The range of values (Q3 to Q1) is the interquartile range (IQR). The whiskers represent Q1–(1.5*IQR) and Q3+(1.5*IQR). The remaining values which do not fall in the range are shown as outliers. (f) Schematic showing the trend of disappearing ORF-free *CEN* neighborhood in the *Clavispora/Candida* clade in Ascomycota. CENP-A^Cse4^ enriched regions are indicated by dark grey bars and the ORF-free regions by the light grey bars. The nature of centromere in each case is also shown. The tree was drawn using PhyloT v2 (https://phylot.biobyte.de/). Download FIG S2, TIF file, 1.0 MB. Copyright © 2021 Narayanan et al. 2021 Narayanan et al. https://creativecommons.org/licenses/by/4.0/ This content is distributed under the terms of the Creative Commons Attribution 4.0 International license.

10.1128/mBio.00905-21.7 TABLE S2 Oligonucleotides used in this study. Download Table S2, DOCX file, 0.02 MB. Copyright © 2021 Narayanan et al. 2021 Narayanan et al. https://creativecommons.org/licenses/by/4.0/ This content is distributed under the terms of the Creative Commons Attribution 4.0 International license.

10.1128/mBio.00905-21.8 TABLE S3 Centromere sequence divergence in different geographical clades. Download Table S3, DOCX file, 0.01 MB. Copyright © 2021 Narayanan et al. 2021 Narayanan et al. https://creativecommons.org/licenses/by/4.0/ This content is distributed under the terms of the Creative Commons Attribution 4.0 International license.

Homology searches for *CEN* sequences among themselves and against the whole genome did not yield any significant results, suggesting that each DNA sequence underlying centromeric chromatin is unique and different. A dot plot comparing each centromere DNA sequence against itself as well as other centromeric sequences suggested the unique nature of sequences and the absence of DNA sequence repeats in C. auris centromeres (Fig. 1e). Searches for specific DNA sequence motifs also did not detect any, except the 40-bp poly(A) and poly(T) stretches, which are present in all the seven regions, though not exclusive to the centromeres (see Fig. S2b). The presence of poly(A) stretches at all centromeres prompted us to analyze the GC content of the *CEN* sequences identified. Two sequence features were investigated using the sliding window approach: GC content (the percentage of G and C residues in the scaffold in a sliding window of 5 kb, with a step size of 1 kb) and GC3 content (GC content at the third position of codons in the annotated ORFs, across the scaffolds, by calculating a moving average of 10 adjacent ORFs). These studies revealed the overlap of C. auris centromere*s* with deep GC and GC3 troughs in all the scaffolds (Fig. 1f).

At each of the seven centromeres in C. auris, core CENP-A^Cse4^ chromatin occupies the entire ORF-free region, often extending partially to the neighboring centromere-proximal ORFs. By comparing the lengths of CENP-A^Cse4^ -bound and the associated ORF-free regions in the previously characterized centromeres of Ascomycota, we observed that centromeric chromatin tends to possess a localized region within the gene-poor zones in species like C. albicans and S. cerevisiae. Exceptionally, the ratio of centromeric chromatin to the remaining ORF-free pericentric region in C. auris, similar to that of C. lusitaniae, is close to 1 (see Fig. S2c). Thus, C. auris, like C. lusitaniae seems to lack pericentric heterochromatin (34). We analyzed RNA-seq data available for C. auris (SRR6900290, SRR6900291, SRR6900292, and SRR6900293) to examine variations of gene expression at the centromere vicinity that might indicate the presence of pericentric heterochromatin. We could not detect any suppression of gene expression in the centromere neighborhoods (see Fig. S2d and e), confirming that C. auris, like C. lusitaniae, possesses pericentric heterochromatin-deficient centromeres (see Fig. S2f). Pericentric heterochromatin formation is a concerted function of pericentric repeats, RNA interference machinery, chromodomain proteins, methyl transferases as well as histone deacetylases. However, these factors have a patchy distribution in the fungal kingdom (35–38). Orthologs of Dcr1 (the noncanonical Dicer protein) are present (B9J08_002318 in C. auris, CXQ85_005187 in *C. haemulonii*, CXQ87_004766 in *C. duobushaemulonii*, and C7M61_003937 in *C. pseudohaemulonii*). However, orthologs of Ago1 (Protein Argonaute), Rdp1 (RNA-dependent RNA polymerase), HP-1 (chromodomain protein), and Clr4 (histone-lysine *N*-methyltransferase) could not be detected in any of these ascomycetes.

### Clade-specific karyotype alterations in *C. auris* involve centromeres.

**FIG 2:**
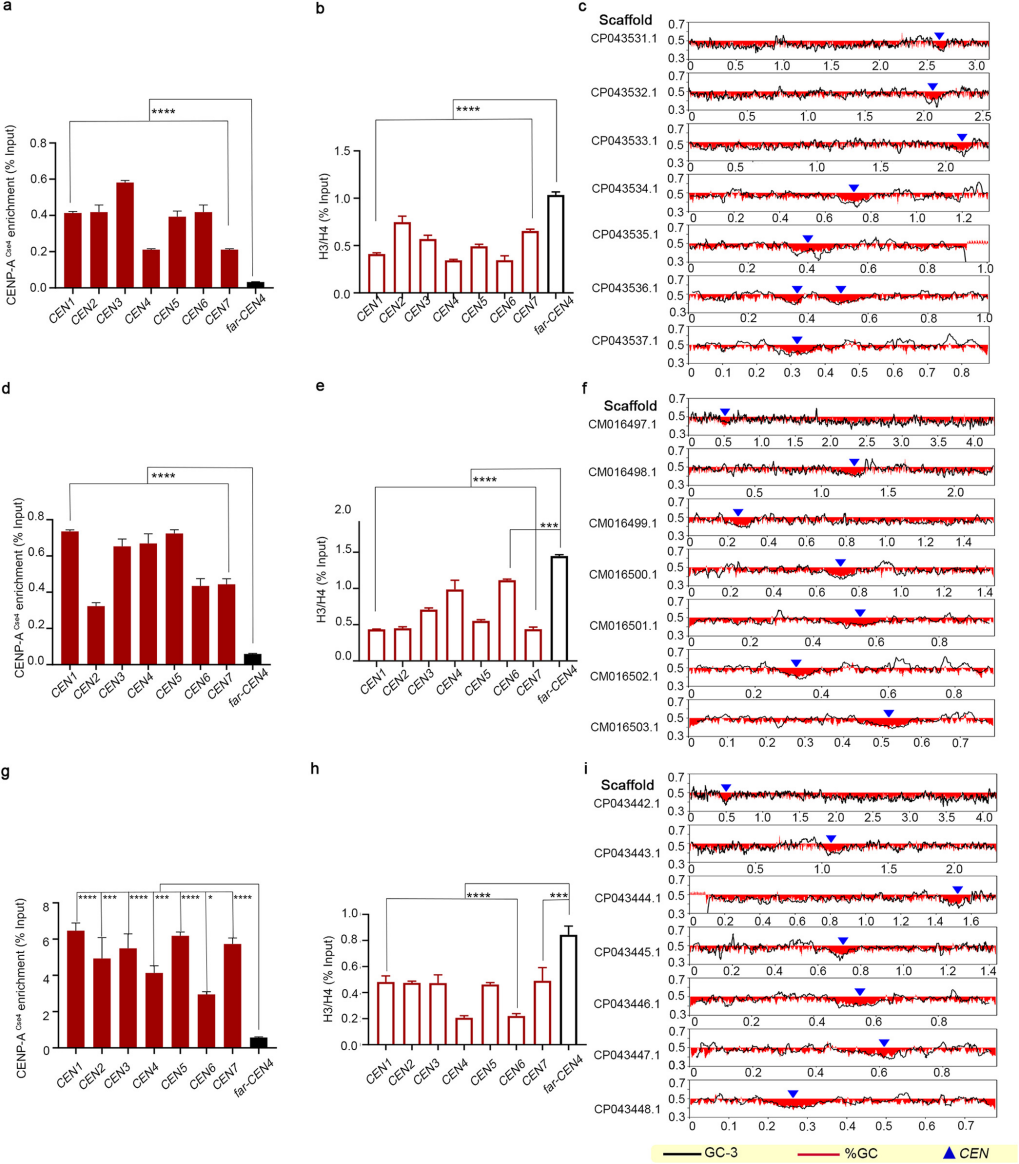
Centromere properties are conserved across the C. auris geographical clades. (a, d, and g) CENP-A^Cse4^ enrichment at the *CEN*s in clade 2 (a), clade 3 (d), and clade 4 (g). The corresponding depletion of canonical histone H3 in clade 2 (b), clade 3 (e), and clade 4 (h) is depicted as H3/H4 ratio on the *y* axis. The percent input values in all the experiments were compared to a control region (*far-CEN4*). qPCR values shown are from three technical replicates. The experiment was repeated twice, with similar results. Error bars indicate standard errors of the mean (SEM). Statistical analysis was done using one-way ANOVA (****, *P* < 0.0001; ***, *P* < 0.001). *CEN* positions (blue triangles) overlap with GC (red) and GC3 (black) scaffold minima in clade 2 (c), clade 3 (f), and clade 4 (i). Coordinates (in Mb) are shown on the *x* axis, and the %GC is shown on the *y* axis. The red bars show the %GC by depicting the amount of deviation from 50% GC (above midline if values are >50% and below if values are <50%). Two copies of the centromere resulting from the detected segmental duplication in clade 2 reference assembly are marked.

**FIG 3:**
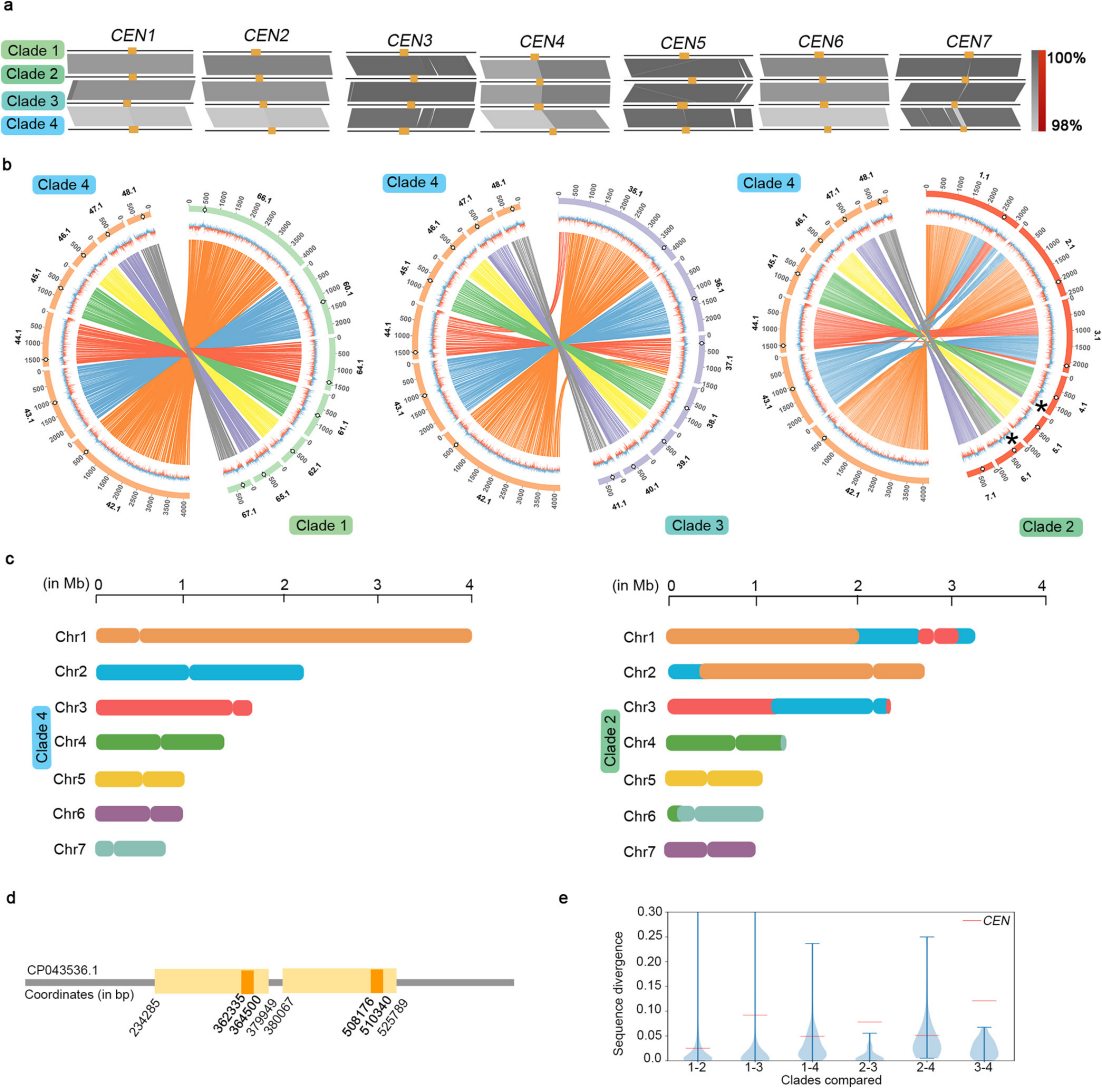
Chromosomal rearrangements resulted in an exclusive centromere relocation in clade 2. (a) Diagram showing immediate *CEN* neighborhood conservation (20 kb each to the left and right of *CEN*s, marked in orange) in each of the four clades. Gray-shaded sections connect homologs; inversions, if present, are indicated in red. The sequence similarity is shown as a percentage. (b) Circos plots showing synteny conservation between different clades. Scaffold numbers are shown on the outermost track with empty circles marking centromere positions, the GC content is shown in the middle track (red, GC content below genome average; blue, AT content above genome average), and the innermost track shows the synteny blocks. A reciprocal translocation event in clade 2 is marked by an asterisk (*). (c) Linear synteny plot showing *CEN* relocations in clade 2 with respect to those of clade 4. *CEN* positions in clades 1 and 3 are similar to clade 4 *CEN* locations. *CEN*s are shown as chromosomal constrictions. Chromosomes are drawn to scale, and chromosomal sizes are shown. (d) Schematic depicting segmental duplication (yellow) in clade 2, placing two copies of the centromere sequences (orange) in the same chromosome in the assembly GCA_003013715.2. The scaffold number and the coordinates are shown. (e) Violin plot depicting divergence at the centromere sequences compared to intergenic regions in each pair of clades.

Clinical isolates of C. auris have been primarily classified into four geographical clades, which exhibit differences in virulence, drug resistance, and genome plasticity (13, 30, 33). Having identified centromeres in a clade 1 isolate, we sought to identify centromere loci in other clades of C. auris. Are the centromeres and their neighborhoods conserved in sequence and location across different geographical clades? To answer this, we predicted the putative centromere coordinates in clades 2, 3, and 4 of C. auris based on gene synteny, GC content, and ORF content using the available assemblies (GCA_003013715.2 of strain B11220 for clade 2, GCA_005234155.1 of strain LOM for clade 3, and GCA_008275145.1 of strain B11245 for clade 4). The predictions were experimentally tested using strains expressing CENP-A^Cse4^–protein A fusion proteins in each of these three clades. The predicted loci were enriched with CENP-A^Cse4^ and depleted of canonical histone H3 (Fig. 2a, b, d, e, g, and h). Like clade 1, all seven identified centromeres in each of the three clades overlap GC and GC3 troughs (Fig. 2c, f, and i). Taken together, we identified small regional AT-rich centromere loci with conserved synteny (Fig. 3a) of all chromosomes in each of the four clades of C. auris.

The genomes of clade 2 and clade 4 have been assembled into seven scaffolds (GenBank assemblies GCA_003013715.2 and GCA_008275145.1, respectively), while the assemblies of clade 1 (GCA_002759435.2) and 3 (GCA_005234155.1) are fragmented. From MLST analysis based on *RPB2* (39), *TUB2*, and *EFB2* gene sequences, we observed that strain A1, isolated in China (SRS4986047), belongs to clade 3 and that strain CA-AM1 (SRS7388889), isolated in Italy, belongs to clade 1. Both GCA_014673535.1 (for strain CA-AM1) and GCA_014217455.1 (for strain A1), being complete assemblies with seven contigs, were used in clade 1 and clade 3 assembly, respectively, for genome-wide comparisons. Centromere locations in these isolates were also identified. Centromere coordinates of all the isolates analyzed are listed in Table 2. Based on the presence of centromeres and syntenic regions shared with CA-AM1, we propose the merger of scaffold PEKT02000002.1 to PEKT02000001.1, PEKT02000005.1 to PEKT02000003.1, and PEKT02000004.1 to PEKT02000007.1 in the current reference assembly of clade 1 to fill the gaps and construct an improved assembly.

**TABLE 2:** Centromeres in four geographical clades of C. auris scaffold map

*CEN*	Scaffold no. (coordinates)^*a*^					
	Clade 1, strain B8441 (GCA_002759435.2)*	Clade 2, strain B11220 (GCA_003013715.2)†	Clade 3, strain LOM (GCA_005234155.1)‡	Clade 4, strain B11245 (GCA_008275145.1)†	Clade 1, strain CA-AM1 (GCA_014673535.1)†	Clade 3, strain A1 (GCA_014217455.1)†
*CEN1*	PEKT02000007.1	CP043532.1 (2073492–2075942)	CM016497.1 (512473–514997)	CP043442.1 (498292–500725)	CP061166.1 (506242–508839)	CP041135.1 (3784930–3787568)
*CEN2*	PEKT02000001.1	CP043533.1 (2125357–2127677)	CM016498.1 (1247622–1249916)	CP043443.1 (1069183–1071379)	CP061160.1 (1241526–1244388)	CP041136.1 (1281760–1284636)
*CEN3*	PEKT02000003.1	CP043531.1 (2624933–2627082)	CM016499.1 (251284–253638)	CP043444.1 (1521159–1524233)	CP061164.1 (1432703–1435251)	CP041137.1 (251504–254010)
*CEN4*	PEKT02000010.1	CP043534.1 (724286–726406)	CM016500.1 (712095–714914)	CP043445.1 (716366–718504)	CP061161.1 (735191–737700)	CP041138.1 (721942–724449)
*CEN5*	PEKT02000009.1	CP043535.1 (398873–401244)	CM016501.1 (546843–550014)	CP043446.1 (547070–549346)	CP061162.1 (550709–553616)	CP041139.1 (447449–450385)
*CEN6*	PEKT02000008.1	CP043537.1 (317145–319270)	CM016502.1 (322203–324715)	CP043447.1 (613673–615766)	CP061165.1 (627643–630375)	CP041140.1 (594422–597157)
*CEN7*	PEKT02000006.1	CP043536.1 (508176–510340, 362335–364500)^*b*^	CM016503.1 (515084–517108)	CP043448.1 (262325–264369)	CP061167.1 (270648–273501)	CP041141.1 (271272–274040)

a *, Incomplete with 15 scaffolds; †, complete with 7 scaffolds; ‡, incomplete with 9 scaffolds.

b Segmental duplication.

Next, we performed genome-wide comparisons using the publicly available chromosome-level assemblies of C. auris to study the involvement of centromeres in clade-specific rearrangements, if any. All combinations of pairwise comparisons revealed interclade chromosomal changes in C. auris. Representative images using clade 4 (GCA_008275145.1) assembly as the reference is shown in Fig. 3b. Centromeres were numbered from 1 to 7 in the clade 4 assembly based on the decreasing sizes of the chromosomes harboring them. Centromeres of clades 1, 2, and 3 were numbered based on synteny with clade 4 *CEN*s. Cross-clade comparisons revealed the genome of clade 2 to be the most rearranged one compared to the other three clades, as reported previously (40) (Fig. 3b). We did not observe any major chromosomal rearrangements between clade 4 and clade 1 assemblies used, while two translocation events were observed between clade 3 and clade 4. Compared to clade 4, five of seven chromosomes in clade 2 had undergone chromosomal rearrangements, resulting in chromosome shuffling. Three of these rearrangements in chromosomes 1,3, and 6 involve synteny breaks near the centromeres (101 kb away from the centromere in chromosome 1, 91 kb away from the centromere in chromosome 3, and 68 kb away from the centromere in chromosome 6). These structural changes resulted in centromere relocations in clade 2 compared to other clades, generating significant karyotype alterations (Fig. 3c). We also detected a segmental duplication in the clade 2 reference assembly (GCA_003013715.2). Duplication of a 145-kb fragment in contig 000006 in the clade 2 assembly places two copies of the centromere region on the same contig, separated by 144 kb (Fig. 3d).

Centromeres were earlier shown to be the most rapidly evolving loci in two closely related species of the CTG-Ser1 clade: Candida albicans and Candida dubliniensis (26). A similar genome-wide analysis among the clades of C. auris suggested that centromeres exhibit high incidence of substitution mutations compared to the intergenic regions of the genome. This is true for all the clades, though the extent of sequence divergence is different (Fig. 3e; see also Table S3). Hence, a geographical clade-specific accelerated evolution of centromere sequences in the same species is evident from these analyses.

### *C. haemulonii* and related species share centromere properties with *C. auris*.

**FIG 4:**
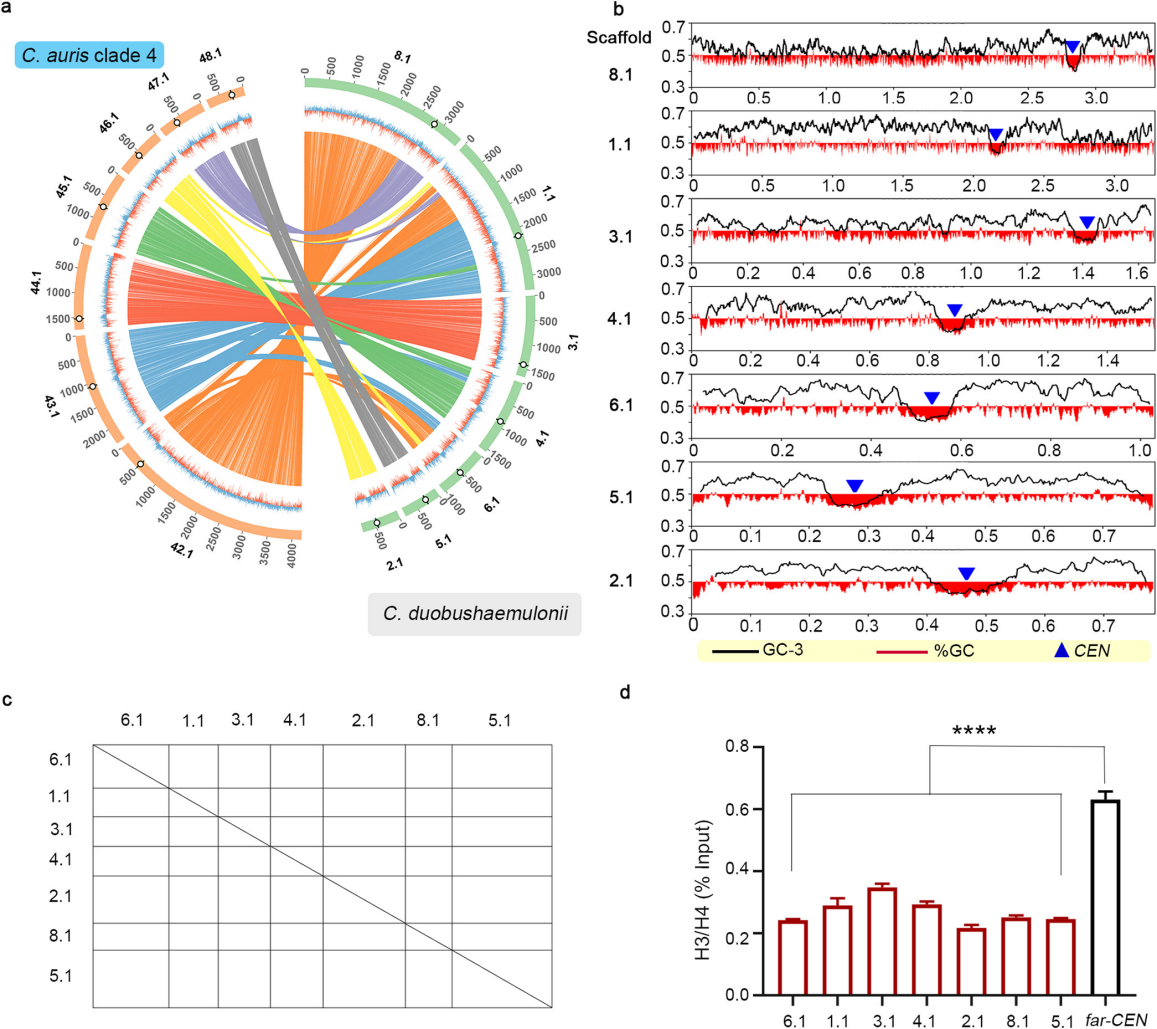
Conservation of centromere properties of the *C. haemulonii* complex species, including C. auris. (a) Loci in *C. duobushaemulonii* chromosome-level assembly syntenic to C. auris *CEN*s. The outermost track of the circos plot depicts genome scaffolds with empty circles marking *CEN* locations, the middle track depicts %GC (red, GC content below genome average; blue, AT content above genome average), and the innermost track shows the synteny blocks. (b) *CEN* positions (blue triangles) overlap with GC (red) and GC3 (black) minima. Coordinates (in Mb) are shown on the *x* axis, and the %GC is shown on the *y* axis. The red color bars show the %GC by depicting the amount of deviation from 50% GC (above midline if values are >50% and below if values are <50%). (c) Dot plot establishing the repeat-free and unique nature of centromere sequences in *C. duobushaemulonii*. The scaffold numbers are shown. (d) Depletion of histone H3 at *CEN*s on different scaffolds (shown on the *x* axis) compared to a noncentromeric control region (far-*CEN*). qPCR values from three technical replicates, represented as percent input, are shown. The experiments were performed three times, with similar results. Error bars indicate standard errors of the mean (SEM). Statistical analysis was performed using one-way ANOVA (****, *P* < 0.0001).

**FIG 5:**
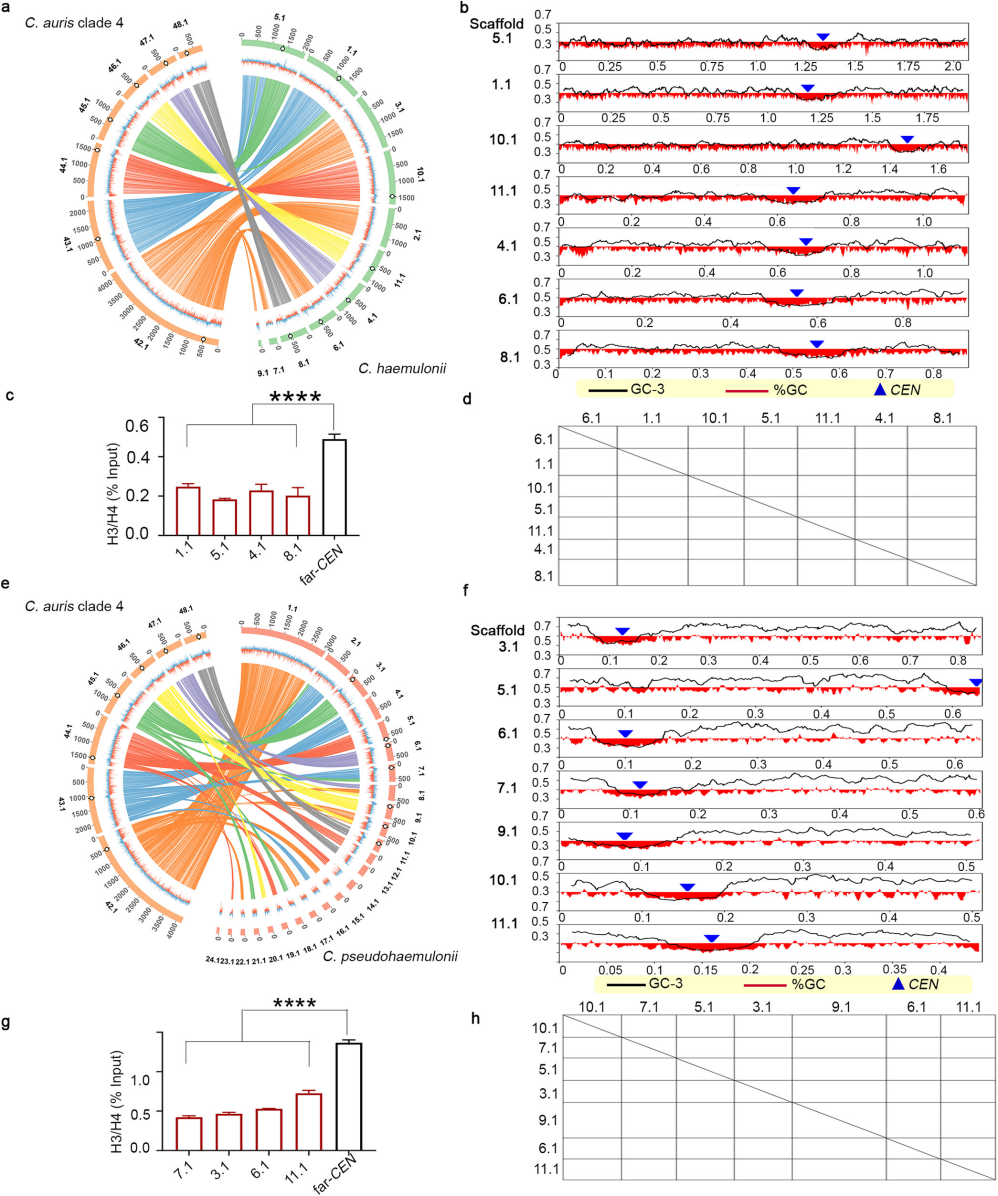
Putative centromeres in *C. haemulonii* and *C. pseudohaemulonii* have properties similar to C. auris centromeres. Circos plots show synteny conservation between C. auris clade 4 and *C. haemulonii* (a) and between C. auris clade 4 and *C. pseudohaemulonii* (e). Genomic scaffolds are shown on the outermost track with the centromere positions marked by empty circles, the middle track shows %GC (red, GC content below genome average; blue, AT content above genome average), and the innermost track depicts synteny blocks. *CEN* positions (blue triangles) overlap with GC (red) and GC3 (black) scaffold minima in *C. haemulonii* (b) and *C. pseudohaemulonii* (f). Coordinates (in Mb) are shown on the *x* axis, and the %GC is shown on the *y* axis. The red color bars show the %GC by depicting the amount of deviation from 50% GC (above midline if values are >50% and below if values are <50%). Histone H3 depletion at the *CEN*s of *C. haemulonii* (c) and *C. pseudohaemulonii* (g) is shown. The percent input values at *CEN*s on different scaffolds (shown on the *x* axis) were compared to a noncentromeric control region (far-*CEN*). The values shown are from three technical replicates, and the experiment was repeated twice, with similar results. Error bars indicate standard errors of the mean (SEM). Statistical analyses were done using one-way ANOVA (****, *P* < 0.0001). Dot plot depicting the uniqueness of *CEN* sequences and absence of repeats in *C. haemulonii* (d) and *C. pseudohaemulonii* (h). Scaffold numbers are shown.

The size of the C. auris genome is 12.2 to 12.4 Mb that falls in the same range with genomes of phylogenetically related, multidrug-resistant, pathogenic species *C. haemulonii*, *C. duobushaemulonii*, and *C. pseudohaemulonii* of sizes 13.3, 12.6, and 12.6 Mb, respectively (based on corresponding NCBI GenBank assemblies; see Materials and Methods). Since all these species of the *C. haemulonii* complex share similar biochemical properties, the misidentification of species in clinics is quite common. Gene synteny around the *CEN* neighborhoods in these species is conserved compared to C. auris, enabling the prediction of *CEN* coordinates (Fig. 4a and Fig. 5a and e). The predicted *CEN* regions were also found to be histone H3 depleted and overlapping with scaffold GC-and GC3 minima (Fig. 4b and c and Fig. 5b, d, f to h), suggesting that these are the bona fide *CEN*s. The identified regions are largely free of ORFs and have lengths comparable to those of C. auris *CEN*s (Table 3). Comparisons utilizing the available chromosome level assembly of *C. duobushaemulonii* revealed that this species has a chromosomal organization more similar to clades 1, 3, and 4 than to clade 2 of C. auris (see Fig. S3a to c), further corroborating the distinctiveness of clade 2, isolates of which are usually drug sensitive.

**TABLE 3:** Centromeres in the *C. haemulonii* complex species scaffold map

C. auris *CEN*	Scaffold no. (coordinates)		
	*C. haemulonii* (GCF_002926055.2)	*C. duobushaemulonii* (GCF_002926085.2)	*C. pseudohaemulonii* (GCF_003013735.1)
*CEN1*	PKFO01000006.1 (551656–554089)	PKFP01000006.1 (533466–536672)	PYFQ01000010.1 (154148–156382)
*CEN2*	PKFO01000001.1 (1190716–1193669)	PKFP01000001.1 (2156789–2158893)	PYFQ01000007.1 (114370–116463)
*CEN3*	PKFO01000010.1 (1471204–1473502)	PKFP01000003.1 (1414893–1417085)	PYFQ01000005.1 (636137–638343)
*CEN4*	PKFO01000005.1 (1337308–1339535)	PKFP01000004.1 (885454–887675)	PYFQ01000003.1 (125155–127358)
*CEN5*	PKFO01000011.1 (642822–645213)	PKFP01000002.1 (465383–468840)	PYFQ01000009.1 (78470–82034)
*CEN6*	PKFO01000004.1 (676456–678623)	PKFP01000008.1 (2827810–2829780)	PYFQ01000006.1 (99229–101295)
*CEN7*	PKFO01000008.1 (549908–552739)	PKFP01000005.1 (275743–279947)	PYFQ01000011.1 (159135–161216)

10.1128/mBio.00905-21.3 FIG S3 Genome-level comparisons reveal the degree of relatedness among species. Circos plot depicting chromosome-level similarities between C. auris clade 1 (strain CA-AM1) and *C. duobushaemulonii* (a), C. auris clade 3 (strain A1) and *C. duobushaemulonii* (b), C. auris clade 2 and *C. duobushaemulonii* (c), and C. auris clade 4 and C. lusitaniae (d). The outermost track shows the genomic scaffolds with the *CEN* positions marked by empty circles, the middle track shows %GC (red, GC content below genome average; blue, AT content above genome average), and the innermost track shows the synteny blocks. Download FIG S3, TIF file, 2.2 MB. Copyright © 2021 Narayanan et al. 2021 Narayanan et al. https://creativecommons.org/licenses/by/4.0/ This content is distributed under the terms of the Creative Commons Attribution 4.0 International license.

### A centromere inactivation event accounts for the chromosome number alteration between *C. lusitaniae* and *C. auris.*

**FIG 6:**
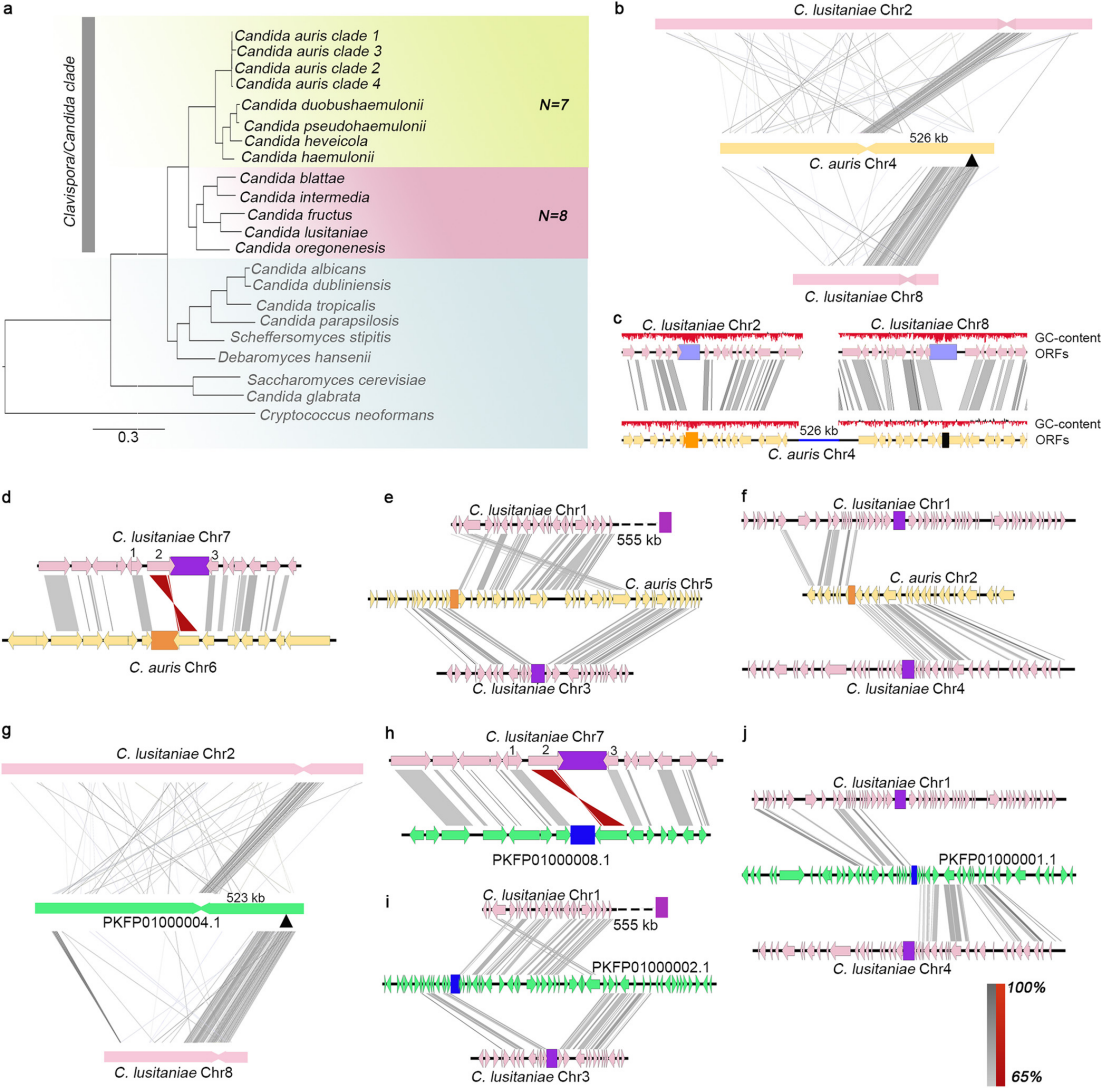
*CEN* inactivation mediated chromosome number variation in C. auris and *C. duobushaemulonii*. (a) Phylogenetic tree depicting the relatedness of C. auris geographical clades and other member species of the *Clavispora/Candida* clade. Other species in Ascomycota with characterized/predicted centromeres are shown. Cryptococcus neoformans (Basidiomycota) are shown as the outgroup. The two chromosome number states detected in *Clavispora/Candida* clade are represented by *n *=* *7 and *n *=* *8. (b) Chromosome-level view depicting the mapping of C. lusitaniae *CEN2* and *CEN8* onto a single scaffold in C. auris. Gray-shaded bars connect homologs; inversions, if present, are indicated in red. Inactive *CEN* (in*CEN*) is shown by a black triangle. The sequence similarity of homologs is shown in the key as a percentage. (c) ORF-level view showing sequence loss and subsequent loss of AT content at in*CEN*. (d) Pericentric inversion in C. auris changing the positions of ORFs 1, 2, and 3, with respect to the C. lusitaniae centromere (purple). C. auris *CEN*s are indicated in orange. (e) Rearrangement involving *CEN*-proximal synteny breaks separating the two synteny blocks on the same chromosome. (f) Synteny breakpoint mapped to the centromere location in C. auris chromosome 2. (g) *CEN* inactivation; (h) pericentric inversion; (i and j) synteny breaks and rearrangements in *C. duobushaemulonii*. *CEN*s are indicated in blue. Inactive *CEN* is shown as a black triangle.

Candida lusitaniae, another opportunistic pathogen, is classified under the *Clavipora/Candida* clade of Metschnikowiaceae and is phylogenetically close to C. auris (Fig. 6a). It was previously reported to have eight AT-rich short regional *CEN*s made up of unique DNA sequences (34). On the other hand, we report that C. auris has seven functional *CEN*s identified in this study. To trace the events that led to the chromosome number reduction during the divergence of these two species, we compared the gene synteny across the centromeres in C. lusitaniae and C. auris. Though the genomes are highly rearranged (see Fig. S3d), we found that the gene synteny around centromeres is conserved between the two species. Intriguingly, chromosome 8 of C. lusitaniae was rearranged as three distinct fragments that fused with other chromosomes of C. auris. As a result, two C. lusitaniae centromeres (*ClCEN2* and *ClCEN8*) were mapped to the same C. auris chromosome, based on synteny analysis (Fig. 6b). ChIP-seq analysis revealed *CEN2* to be functional in C. auris out of the two regions as CENP-A^Cse4^ is recruited only at *CEN2*. This observation illustrates a clear example of “evolution in progress” as the region corresponding to C. lusitaniae *CEN8* becomes nonfunctional in C. auris despite gene synteny conservation between the two species around this region. *ClCEN8*, the functional centromere of chromosome 8 in C. lusitaniae, spans a region of ∼4.5 kb, while the average centromere length is 4.3 kb. The size of the corresponding syntenic regions of the inactivated centromere (in*CEN*) is 1.1 kb in C. auris. In comparison, the functional centromeres of the same species have an average length of 2.7 kb. We posit that the significant, centromere-specific attrition of DNA sequence accompanied by the reduction of AT-content resulted in the centromere inactivation in C. auris (Fig. 6c). Analysis at the sequence level reveals divergence at the in*CEN* to be intermediate of that of centromeres and intergenic regions, further suggesting a “transition from centromeric to intergenic region” (see Table S3).

A distinct *CEN*-associated structural change observed in C. auris, compared to the syntenic *CEN* in C. lusitaniae, is a pericentric inversion altering the relative positions of three ORFs (Fig. 6d). In addition to the presence of in*CEN*, five centromere regions in C. lusitaniae (*ClCEN1*, *ClCEN2*, *ClCEN5*, *ClCEN6*, and *ClCEN7*) have syntenic centromeres in C. auris. The remaining two centromeres of C. auris identified through CENP-A^Cse4^ ChIP-seq are located at synteny breakpoints. The immediate ORFs flanking *CEN3* in C. lusitaniae are conserved in C. auris but are separated by a length of 55 kb. The centromere is located adjacent to one of the synteny blocks, resulting in partial synteny conservation (Fig. 6e). We also mapped a synteny breakpoint at the centromere on chromosome 2 of C. auris. The ORFs on either side of the C. auris *CEN2* maps to different chromosomes in C. lusitaniae (Fig. 6f).

The same patterns were observed in *C. haemulonii*, *C. duobushaemulonii*, and *C. pseudohaemulonii*, where sequences syntenic to *ClCEN8*-flanking blocks map to the same scaffold bearing *ClCEN2* synteny regions (Fig. 6g; see also Fig. S4a and b). The region corresponding to *ClCEN8* has undergone differential sequence attrition in these species, resulting in reduced sequence length (840 bp in *C. haemulonii*, 361 bp in *C. duobushaemulonii*, and 496 bp in *C. pseudohaemulonii*) as observed in C. auris in*CEN*. *CEN*-specific sequence loss has also resulted in the reduction of AT-content in these species. *CEN*-associated inversions and synteny breakpoints in these species are also identical to those in C. auris (Fig. 6h to j; see also Fig. S4c to h). The typical patterns of *CEN*-associated changes in C. auris and other species of the *C. haemulonii* complex suggest that these events must have occurred in an immediate common ancestor before species divergence.

10.1128/mBio.00905-21.4 FIG S4 Centromere-associated structural changes in *C. haemulonii* and *C. pseudohaemulonii*, compared to C. lusitaniae, are similar to those in C. auris. (a and b) Mapping of C. lusitaniae *CEN2* and *CEN8* synteny blocks onto the same scaffold in *C. haemulonii* (a) and *C. pseudohaemulonii* (b). Inactive *CEN* is indicated by black triangles. (c and d) Pericentric inversion changing the relative position of ORFs 1, 2, and 3 in *C. haemulonii* (c) and *C. pseudohaemulonii* (d). (e and f) Separation of synteny blocks flanking C. lusitaniae *CEN3* by ∼55 kb on the same chromosome in *C. haemulonii* (e) and *C. pseudohaemulonii* (f). (g and h) Mapping of a synteny breakpoint at the centromere in *C. haemulonii* (g) and *C. pseudohaemulonii* (h). Centromeres in C. lusitaniae are marked by boxes (purple, C. lusitaniae; brown, *C. haemulonii*, and green, *C. pseudohaemulonii*). Gray shading connects homologs, and inversions (if present) are indicated in red. The sequence similarity is shown as a percentage in the key. Download FIG S4, TIF file, 2.2 MB. Copyright © 2021 Narayanan et al. 2021 Narayanan et al. https://creativecommons.org/licenses/by/4.0/ This content is distributed under the terms of the Creative Commons Attribution 4.0 International license.

### Putative small regional, AT-rich centromeres identified in other species of the *Clavispora/Candida* clade.

**FIG 7:**
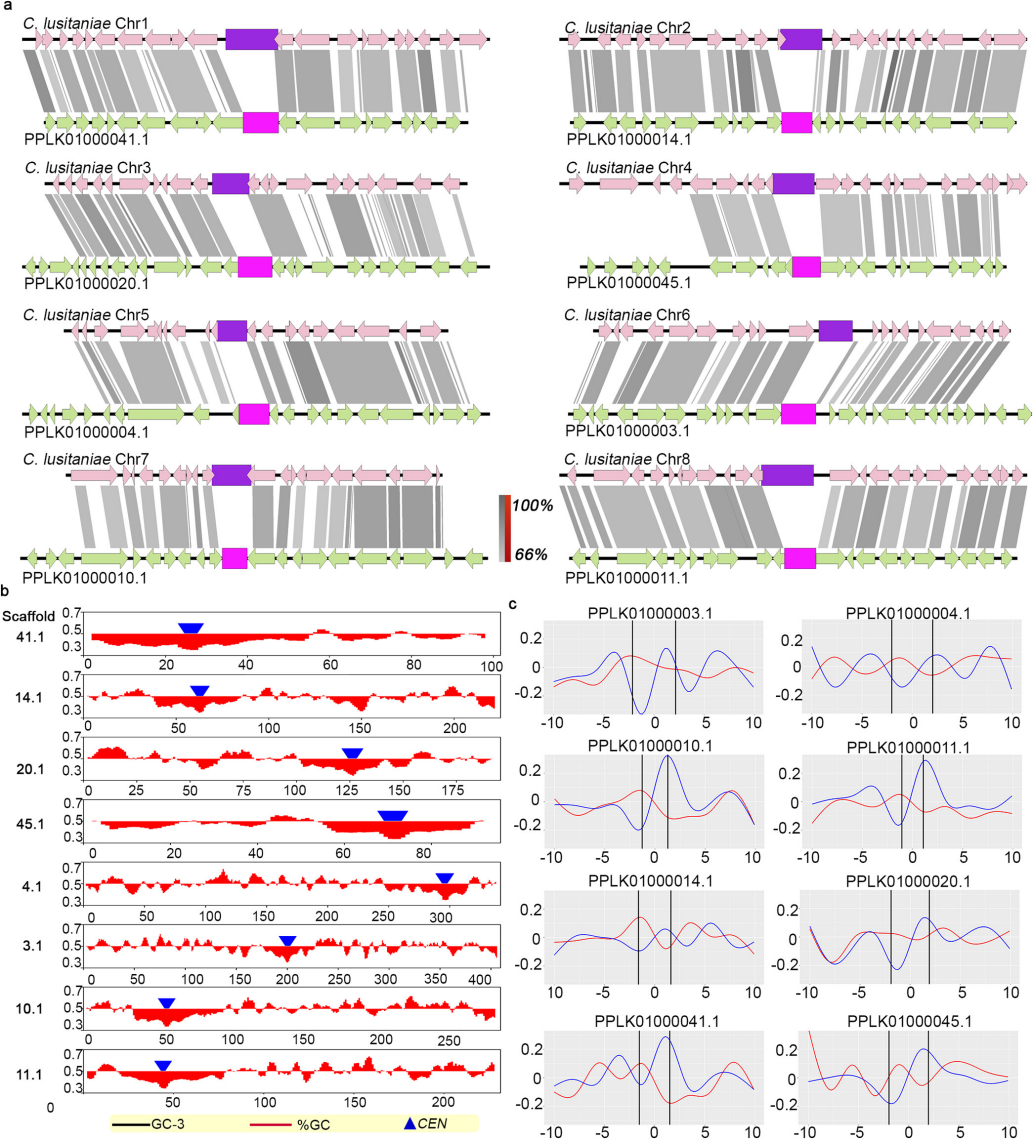
Eight putative centromeres identified in *C. fructus*. (a) Eight loci in *C. fructus* syntenic to C. lusitaniae centromeres (purple). Putative *CEN*s in *C. fructus* are indicated in pink. Gray shading connects homologs; inversions (if present) are indicated in red. The sequence similarity is shown as a percentage in the key. (b) Putative *CEN* positions (blue triangles) overlap with GC (red) and GC3 (black) scaffold minima. Coordinates (in Mb) are shown on the *x* axis, and the %GC is shown on the *y* axis. (c) Plots showing the presence of GC-skews (blue) and AT-skews (red) at the putative *CEN*s (‖). Distance from *CEN* (in kb) is shown on the *x* axis, and the skew is shown on the *y* axis. The red color bars show the %GC by depicting the amount of deviation from 50% GC (above midline if values are >50% and below if values are <50%).

Around 40 ascomycetous species are classified under the *Clavispora/Candida* clade of Metschnikowiaceae (41). To explore the centromere properties in the *Clavispora/Candida* clade, we attempted *CEN* identification in other species for which genome assemblies are available (Fig. 6a). We could locate putative centromeres in several fungal species of the *Clavispora/Candida* clade of Metschnikowiaceae based on the conserved gene synteny and other conserved centromere properties of C. auris and C. lusitaniae as references (see Table S4). Two possible chromosome number states were detected in the *Clavispora/Candida* clade, and the analyzed genomes were classified into two groups: (i) species which have eight AT-rich putative centromeric loci of comparable sizes and (ii) species with seven AT-rich putative centromeric loci with an eighth locus that had undergone sequence loss despite synteny conservation around the orthologous but presumably inactivated centromere locus. C. lusitaniae has eight AT-rich, ORF-free centromeres of comparable lengths. *Candida fructus* was found to possess eight loci syntenic to each of the eight centromeres in C. lusitaniae. The identified regions are also depleted of ORFs, are GC-poor, and harbor GC skews as reported in the case of C. lusitaniae and C. albicans centromeres (34, 42) (Fig. 7). Each of C. auris, other species of the *C. haemulonii* complex, and *Candida heveicola* has seven ORF-free loci, which are GC-poor. The eighth locus, though syntenic to *CEN8* of C. lusitaniae, has undergone sequence attrition in each of them and is likely to be inactive, like the in*CEN* of C. auris. We could identify loci in other related species, including Candida intermedia, *Candida blattae*, and *Candida oregonensis* syntenic to each of the seven centromeres of C. auris. All the predicted regions are ORF-free, AT-rich, and constituted by unique, repeat-free sequences (see Fig. S5a and b). We also identified an eighth locus syntenic to C. lusitaniae *CEN8* in these species. Unlike the in*CEN* in C. auris with a drastically reduced sequence length, the eighth locus is of similar size as other predicted centromeres in these three species (see Fig. S5a and c). The conservation of sequence length suggests that they may have eight functional centromeres. Exceptionally due to a possible assembly error, two putative centromeres identified in *C. intermedia* map to the same scaffold. Our *in silico* analyses collectively suggest the existence of two chromosome number states and remarkably similar centromere properties shared by these closely related organisms of the *Clavispora/Candida* clade. While all these putative *CEN* loci show similar gene synteny, ORF abundance, sequence length, and GC content, further experimental validation is required before assigning them as authentic *CEN* loci of the respective organisms.

10.1128/mBio.00905-21.5 FIG S5 Conserved *CEN* neighborhoods in other species of the *Clavispora/Candida* clade. (a) The Locations of *CEN*s in *C. blattae*, *C. intermedia*, *C. heveicola*, and *C. oregonensis* were predicted based on gene synteny conservation, using C. auris as the reference. The color code for ORFs and putative *CEN*s for each species is given in the key. Blue indicates the GC content, gray shading connects homologs, and inversions (if present) are indicated in red. The sequence similarity is shown as a percentage in the key. (b) Dot plots showing the repeat-free, unique sequences at the centromeres in different species. Centromeres are numbered using C. auris as the reference. (c) Plot comparing the lengths of centromeres identified/predicted in the study. Lengths of CENP-A^Cse4^-enriched regions are shown for C. lusitaniae and C. auris. The lengths of the ORF-free regions are shown for the rest of the species. The inactive *CEN* is depicted in black. Download FIG S5, TIF file, 1.2 MB. Copyright © 2021 Narayanan et al. 2021 Narayanan et al. https://creativecommons.org/licenses/by/4.0/ This content is distributed under the terms of the Creative Commons Attribution 4.0 International license.

10.1128/mBio.00905-21.9 TABLE S4 Centromeres in related species scaffold map. Download Table S4, DOCX file, 0.01 MB. Copyright © 2021 Narayanan et al. 2021 Narayanan et al. https://creativecommons.org/licenses/by/4.0/ This content is distributed under the terms of the Creative Commons Attribution 4.0 International license.

### Clade 2 of *C. auris* follows a unique evolutionary trajectory.

**FIG 8:**
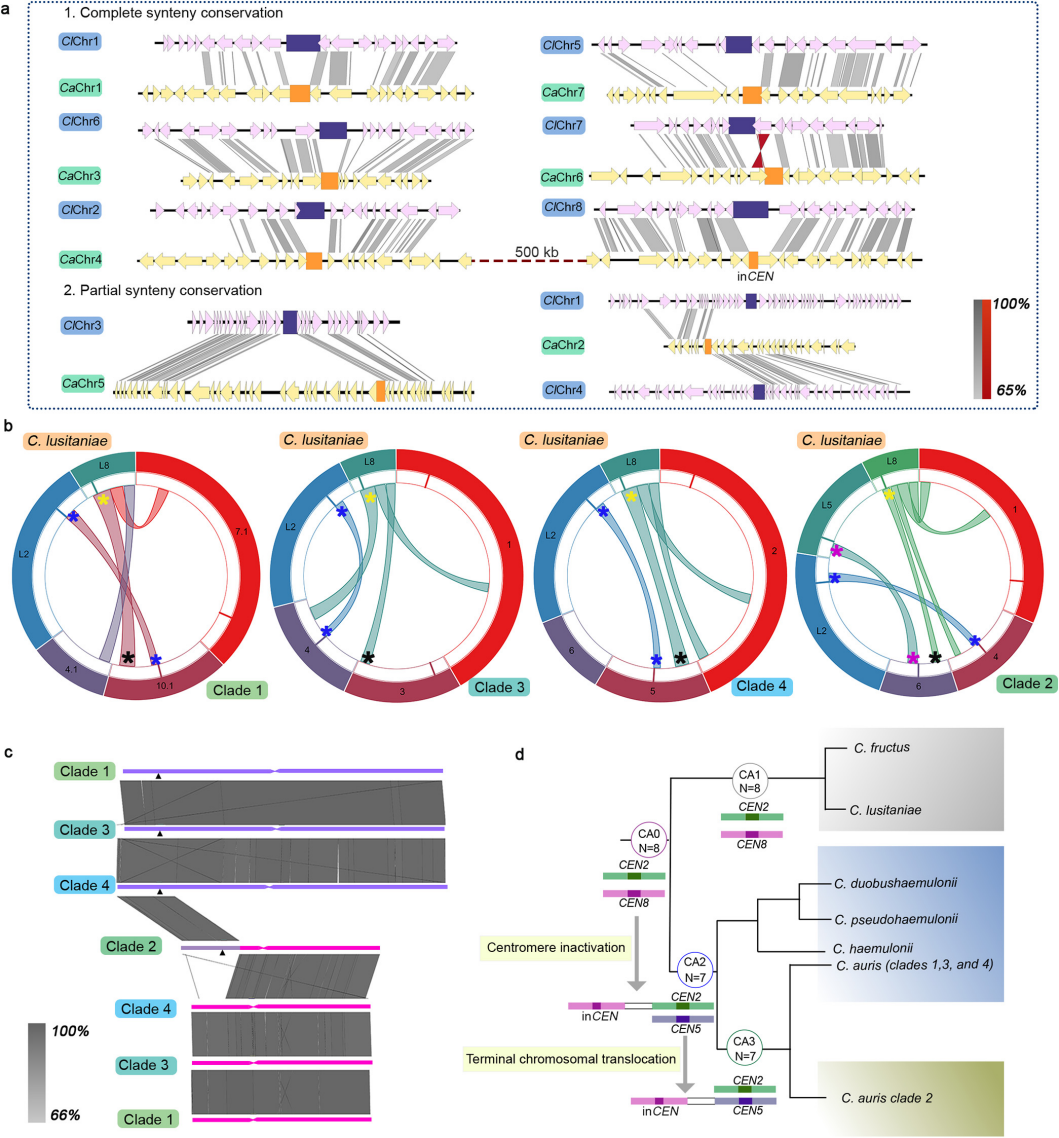
C. auris clade 2 evolved via a unique evolutionary trajectory. (a) Representative genome of the common ancestor of C. auris and the *C. haemulonii* complex species (*n *=* *7), depicting chromosomal rearrangement patterns with respect to the common ancestor of C. lusitaniae and *C. fructus* (*n *=* *8). *CEN*s in the common ancestor (*n* = 8, chromosomes shown as *Cl*Chr) are indicated in purple, and *CEN*s in the common ancestor (*n *=* *7, chromosomes shown as *Ca*Chr) are indicated in orange. Homologs are connected by gray shading, and inversions, if present, are indicated in red. The sequence similarity is shown as a percentage in the key. (b) C. lusitaniae *CEN*2 (on Chr2, L2) and *CEN8* (on Chr8, L8) map to the same scaffold in C. auris clades 1, 3, and 4, whereas *CEN5* (on Chr5, L5) and *CEN8* map to the same scaffold in C. auris clade 2. Corresponding genomic scaffolds are shown in the outermost track, and the synteny blocks are depicted in the innermost track. The inactive centromere is marked in black and the corresponding active centromere in yellow. (c) Terminal chromosomal translocation event resulting in the relocation of in*CEN* (black triangles) in clade 2. Constrictions mark *CEN*s syntenic to C. lusitaniae *CEN2* and *CEN5*. Sequence similarity is shown as a percentage in the key. (d) A *CEN*-based model tracing the event of centromere inactivation in the common ancestor CA0, resulting in chromosome number reduction in CA2, while CA1 maintains the chromosome number of 8. CA2 represents the common ancestor of C. auris and *C. haemulonii* complex, and CA3 is the common ancestor of all C. auris clades. A TCT event further repositions the inactive *CEN* in clade 2, representing the unique evolutionary trajectory of C. auris clade 2. *CEN*s are numbered using C. lusitaniae as the reference.

We posit that C. lusitaniae and *C. fructus* might have shared an immediate common ancestor CA1 with eight functional *CEN*s, one on each chromosome (*n *=* *8). Chromosomal rearrangements placed regions syntenic to *ClCEN2* and *ClCEN8* of these two species on the same chromosome in the *C. haemulonii* complex species as well as three clades (clades 1, 3, and 4) of C. auris, out of which *ClCEN2* is active, and *ClCEN8* is inactive (in*CEN*) (Fig. 8a). This finding indicates the existence of an immediate common ancestor (*n = 7*), CA2, with a *ClCEN2*-in*CEN* configuration shared by C. auris and other species of the *C. haemulonii* complex. Synteny analyses enabled us to reconstruct *CEN*-based ancestral genomes of the immediate common ancestors of C. lusitaniae-*C. fructus* (CA1) and *C. haemulonii* complex-C. auris (CA2), representing chromosome number states of *n = 8* and *n = 7*, respectively (Fig. 8a). We also hypothesize parallel evolution of the geographical clades of C. auris, at different time scales, diverging from a common ancestor CA3, which was derived from the ancestor CA2. Out of the four clades, clade 2 has a remarkably rearranged genome. The location of in*CEN* serves as a useful index for representing interclade differences. The synteny block containing C. lusitaniae *CEN8* is conserved in *C. haemulonii*, *C. pseudohaemulonii*, and *C. duobushaemulonii*, as well as in C. auris clades 1, 3, and 4. The genes in the block are found distributed in two chromosomes in clade 2, indicating that a break occurred within the block, followed by a downstream reciprocal translocation event (Fig. 3b; see also Table S5). The terminal chromosomal translocation (TCT) event in which Chr4 and Chr7 of CA3 exchanged chromosome ends might have repositioned in*CEN* resulting in a *ClCEN5-*in*CEN* configuration (Fig. 3b and Fig. 8b), exclusive to clade 2. This structural change further confirms the divergence of clade 2 from the common ancestor CA3 along a different evolutionary trajectory (Fig. 8c). On analyzing the whole-genome synteny conservation, we observed that the chromosomes of clade 2 are more rearranged with respect to *C. duobushaemulonii* chromosomes, compared to the chromosomes of the other clades (see Fig. S3), supporting the inference that clade 2 is uniquely rearranged. Also, the conservation of the C. lusitaniae *CEN8*-containing synteny block among the *C. haemulonii* complex species and all of the C. auris clades except clade 2 further suggests that clade 2 underwent major karyotype changes different from all the other clades and related species. These observations prompted us to reject an equally possible, alternative model of clade 2 being the ancestral unique strain where the event leading to chromosome number reduction happened. In this case, clade 2 would have shared higher similarity with C. lusitaniae with respect to the synteny block harboring in*CEN*. Other rearrangements causing *CEN* relocations provide additional lines of evidence for the clade-specific divergence.

10.1128/mBio.00905-21.10 TABLE S5 Distribution of C. lusitaniae *CEN8*-containing synteny block in C. auris clades and related species. Download Table S5, DOCX file, 0.02 MB. Copyright © 2021 Narayanan et al. 2021 Narayanan et al. https://creativecommons.org/licenses/by/4.0/ This content is distributed under the terms of the Creative Commons Attribution 4.0 International license.

## DISCUSSION

Centromere identification revealed a typical centromere landscape in multiple species of the *Clavispora/Candida* clade—small regional *CEN*s constituted by AT-rich unique sequences and embedded in ORF-free regions that are devoid of any detectable pericentric heterochromatin, DNA motifs, or repeats. These closely related species either contain seven chromosomes or eight chromosomes. We propose that a centromere inactivation event in a common ancestor with eight chromosomes led to this diversity. The inactive centromere, in a pseudodicentric chromosome that might have formed at an intermediate stage, underwent substantial but differential attrition of centromere DNA sequence. This process might have played a crucial role in the emergence of multiple species with seven chromosomes. Inactivation of centromere function mediated by DNA sequence deletion has been suggested previously (43–45). Several synteny breakpoints mapped to the identified centromeres, compared to representative species of the eight-chromosome state, add to the growing evidence that suggests centromeres as a hub of fragility in different systems (46, 47) and downstream chromosomal rearrangements. Spatial proximity of clustered centromeres in fungal species facilitates intercentromeric recombination, possibly mediated by replication fork stalling and higher chances of double-stranded breaks, thus contributing toward karyotype evolution (17, 48, 49). The role of AT-rich sequences and poly(A) stretches in these events, owing to their melting features and potential propensity to form non-B DNA, warrants further study as centromeres in many fungal species coincide with GC or GC3 troughs (16, 28, 50–53).

Whole chromosome and segmental aneuploidy are correlated with drug resistance in other fungal pathogens (54). The C. auris genome is known to be highly plastic (33). Considering the multidrug resistance and karyotype plasticity of C. auris, it is likely that gross chromosomal rearrangements are taking place in different clinical isolates, contributing to their drug resistance or virulence. Mapping of centromere loci should help trace such genomic rearrangement events. Centromere sequences in different geographical clades were found to evolve rapidly and differentially than the rest of the genome, suggesting that centromeres are potential candidate loci to study evolutionary trajectories emerging within a species. C. auris clade 2 has the most rearranged genome and consists of atypical isolates that differ from the other clades in terms of drug tolerance, as well as pathogenicity (40, 55, 56). The unique nature of centromere sequences can be used for accurate species-level and clade-level identification.

In this study, we reveal that the genome of clade 2 differs from the rest of the clades in the position of orthologous centromeres on the chromosomes and the location of the inactive centromere. Chromosome-level comparisons also reveal that karyotype of clade 2 is more rearranged and hence different from *C. duobushaemulonii* than the other clades. These observations directed us to conclude that C. auris clades diverged from a common ancestor that shares ancestry with the *C. haemulonii* complex species, and from which clade 2 diverged along a different trajectory during the parallel evolution of the geographical clades. Significant karyotype alterations, evident from the centromere and inactive centromere locations are likely to have contributed to the distinctiveness of C. auris clade2, compared to other clades and the *C. haemulonii* complex species. Ascomycetous pathogens such as C. albicans and C. glabrata exist as clades that exhibit geographical specificity and clade-specific phenotypic features (57, 58). Rare or no interclade recombination is observed in these species, and little is known about the genomic rearrangements or the variations at centromeres operating at the clade level, which can, in turn, affect the recombination frequency.

We conjecture that such centromere-associated clade-specific differences might not be restricted to C. auris. Further exploration of centromere sequences and associated structural changes within a species and species complexes will yield deeper insight into the role of centromeres in generating diversity in primarily asexual fungi.

## MATERIALS AND METHODS

### Strains, media, and growth conditions.

Strains of various *Candida* species used in the study (listed in Table S1 in the supplemental material) were grown in YPD (1% yeast extract, 2% peptone, and 2% dextrose) at 30°C. The identity of the strains was confirmed by amplification and sequencing of the internal transcribed spacer (ITS) and D1/D2 regions, followed by BLAST analysis (http://www.ncbi.nlm.nih.gov/BLAST/Blast.cgi). The clade status of different C. auris isolates used was confirmed by amplifying and sequencing regions of three housekeeping genes (*TUB2*, *EFB1*, and *RPB1*) harboring polymorphic sites (*TUB2*, bp 534; *EFB1*, bp 698; and *RPB1*, bp 552 [with respect to clade 1]).

10.1128/mBio.00905-21.6 TABLE S1 Strains used in this study. Download Table S1, DOCX file, 0.01 MB. Copyright © 2021 Narayanan et al. 2021 Narayanan et al. https://creativecommons.org/licenses/by/4.0/ This content is distributed under the terms of the Creative Commons Attribution 4.0 International license.

### Construction of *C. auris* strain expressing CENP-A^Cse4^–protein A fusion protein.

The homolog of CENP-A^Cse4^ in C. auris was identified by BLAST using C. albicans CENP-A^Cse4^ sequence as the query against the C. auris genome. It was distinguished from the canonical histone H3 sequences by confirming the presence of CENP-A^Cse4^-specific amino acid residues (59). For tagging CENP-A^Cse4^ with protein A at the C terminus, approximately 900 and 800 bp were used as upstream and downstream sequences, respectively, to construct the tagging cassette. The 900-bp fragment (including the complete ORF and native promoter sequence) was amplified from the genomic DNA and cloned as a KpnI-SacI fragment in the pBS-TAP-NAT plasmid. The downstream sequence was cloned as a SpeI-NotI fragment. The 3.7-kb tagging construct, as a KpnI-NotI fragment, was used to transform Cau46R. The transformation of the strains was performed as previously described (60). Nourseothricin (Jena Bioscience) was added at a concentration of 100 μg/ml in the media for selecting transformants. The colonies obtained were subcultured in the presence of nourseothricin and integration of the tagging construct in NAT^+^ transformants was confirmed by PCR.

### Western blotting.

Cells were grown overnight in YPD until mid-log phase, and 3 optical density (OD) equivalent cells were harvested for protein lysate preparation. The cells were suspended in 400 μl of ice-cold trichloroacetic acid (12.5%), vortexed briefly, and stored at −20°C overnight. The samples were later thawed and pelleted by centrifugation at 14,000 rpm at 4°C for 10 min. The pellets were washed twice with 400 μl of ice-cold acetone (80%), air-dried, suspended in an appropriate volume of lysis buffer (0.1 M NaOH and 1% SDS), and boiled for 10 min. The proteins in the lysate were separated on 12% polyacrylamide gels. The separated samples were transferred from the gels to the nitrocellulose membranes, which were then probed with anti-protein A antibodies (Sigma, P3775; 1:5,000 dilution in 2.5% [wt/vol] skim milk powder in 1× PBS) and horseradish peroxidase-conjugated goat anti-rabbit secondary antibodies (Abcam, 1:10,000 dilution in 2.5% [wt/vol] skim milk powder in 1× PBS). The blots were developed using Chemiluminescence Ultra substrate (Bio-Rad) and imaged using the VersaDoc system (Bio-Rad).

### Preparation of spheroplasts.

Cells were grown in 50 ml of YPD until reaching an optical density at 600 nm (OD_600_) of 0.8 and washed with water by centrifugation at 3,000 rpm for 5 min. Cells were then incubated in 10 ml of 2-mercaptoethanol solution (5% in water; Himedia, catalog no. MB041) for 1 h at 30°C at 180 rpm. The cells were pelleted, washed, and resuspended in SCE buffer (1 M sorbitol, 100 mM sodium citrate, 10 mM EDTA [pH 8.0]). Lysing enzyme from *Trichoderma harzianum* (Sigma, catalog no. L1412) was added at a concentration of 2.5 mg/ml, and the suspension was incubated at 37°C at 80 rpm for 2 h. The cells were examined under a microscope to determine the proportion of spheroplasts in the suspension. The prepared spheroplasts were further processed based on the corresponding experimental design.

### Indirect immunofluorescence.

The C. auris CENP-A^Cse4^–protein A strain was inoculated to 1% (vol/vol) from an overnight culture and was grown until reaching an OD_600_ of 0.8. The cells were fixed by adding formaldehyde to a final concentration of 1% for 15 min. Spheroplasts were prepared from the fixed cells (as described above), washed with 1× PBS, and diluted in 1× PBS to a density appropriate for microscopy. Slides for microscopy were washed and coated with poly l-lysine (10 mg/ml). Portions (20 μl) of the diluted cell suspension were added onto slides, followed by incubation at room temperature for 5 min. The suspension was aspirated, and the slide was washed to remove unbound spheroplasts. The slide was treated with ice-cold methanol for 6 min, followed by ice-cold acetone for 30 s. Blocking solution (2% nonfat skim milk powder in 1× PBS) was added to each well, and the slide was incubated for 30 min at room temperature. The blocking solution was aspirated, and rabbit anti-protein A antibodies (Sigma, P3775; dilution, 1:1,000) were added. The slide was incubated in a wet chamber for 1 h. The antibodies were aspirated, and the slide was washed 15 times, incubating the slide for 2 min for each wash. Secondary antibodies were added (Alexa Fluor 568-goat anti-rabbit IgG; Invitrogen, A11011; dilution, 1:1,000). The slide was incubated in the dark in a wet chamber for 1 h at room temperature. The washes were repeated, and mounting medium (70% glycerol with 100 ng/ml DAPI [4′,6′-diamidino-2-phenylindole]) was added. Clean coverslips were mounted onto the wells, and the slides were imaged using an inverted fluorescence microscope (Zeiss Axio observer; Plan Apochromat, 100× oil). Images were processed using Zeiss ZEN system software and ImageJ.

### Chromatin immunoprecipitation.

C. auris CENP-A^Cse4^–protein A strain was inoculated to 1% (vol/vol) from an overnight culture, grown until reaching an OD_600_ of 1.0, and cross-linked by the addition of formaldehyde to a final concentration of 1% for 15 min. Quenching with 0.135 mM glycine for 5 min was followed by preparation of spheroplasts (as described above). The following buffers were used to wash the prepared spheroplasts: 1× PBS (ice-cold), Buffer-1 (0.25% Triton X-100, 10 mM EDTA, 0.5 mM EGTA, 10 mM Na-HEPES [pH 6.5]), and Buffer-2 (200 mM NaCl, 1 mM EDTA, 0.5 mM EGTA, 10 mM Na-HEPES [pH 6.5]). Then, 1 ml of lysis buffer (50 mM HEPES [pH 7.4], 1% Triton X-100, 140 mM NaCl, 0.1% sodium deoxycholate, 1 mM EDTA) was added to the pellet obtained after the final wash, along with protease inhibitor cocktail (1×). The resuspended spheroplasts were sonicated to obtain chromatin fragments in the size range of 100 to 400 bp. The lysate was cleared by centrifugation at 14,000 rpm for 10 min at 4°C. One-tenth of the lysate volume was separated to be used as the input DNA. The remaining lysate was divided into two equal fractions: anti-protein A antibodies were added to one of the fractions (immunoprecipitation [IP] fraction) at a 20-μg/ml concentration. The other fraction served as the antibody-minus control. Both the fractions were incubated overnight on a Rotaspin at 4°C. Protein A-Sepharose beads were added, and the samples were incubated on a Rotaspin at 4°C for 6 h. This was followed by collecting the beads by centrifugation and sequential washes with the following buffers: twice with 1 ml of low-salt wash buffer (0.1% SDS, 1% Triton X-100, 2 mM EDTA, 20 mM Tris [pH 8.0], 150 mM NaCl), twice with 1 ml of high-salt wash buffer (0.1% SDS, 1% Triton X-100, 2 mM EDTA, 20 mM Tris [pH 8.0], 500 mM NaCl), once with 1 ml of LiCl wash buffer (0.25 M LiCl, 1% NP-40, 1% sodium deoxycholate, 1 mM EDTA, 10 mM Tris [pH 8.0]), and twice with 1 ml of 1× Tris-EDTA (10 mM Tris [pH 8.0], 1 mM EDTA). For each wash, the beads were rotated on a Rotaspin for 5 min in the corresponding buffer, followed by centrifugation at 5,400 rpm for 2 min. The beads were suspended in 0.25 ml of elution buffer (0.1 M NaHCO_3_, 1% SDS), incubated at 65°C for 5 min, and rotated on the Rotaspin for 15 min. The supernatant was collected after centrifugation. The elution step was repeated to obtain a final eluted volume of 0.5 ml. The elution buffer was also added to the stored input sample to obtain a final volume of 0.5 ml. Decrosslinking of the three samples (input, IP, and antibody-minus) was done by adding 20 μl of 5 M NaCl and overnight incubation at 65°C. Proteins in the samples were removed by adding 10 μl of 0.5 M EDTA, 20 μl of 1 M Tris (pH 6.8), and 2 μl of proteinase K (20 mg/liter), followed by incubation at 45°C for 3 h. An equal volume of phenol-chloroform-isoamyl alcohol (25:24:1) was added to purify the samples, and the aqueous phase was extracted by centrifugation at 14,000 rpm for 10 min. DNA was precipitated by adding 3 M sodium acetate (1/10th of the volume [pH 5.2]), 1 μl of glycogen (20 mg/ml), and 1 ml of absolute ethanol, followed by incubation at −20°C overnight. The precipitated DNA was collected by centrifugation at 13,000 rpm for 30 min at 4°C and was washed once with 70% ethanol. Air-dried pellets were resuspended in 20 μl of sterile MilliQ water with 10 mg/ml RNase. ChIP-DNA from duplicates were pooled for ChIP-seq.

The same protocol was followed to determine canonical histone H3 and histone H4 occupancy at the centromeres in *C. haemulonii*, *C. duobushaemulonii*, *C. pseudohaemulonii*, and different clades of C. auris, with some differences. Anti-H3 antibodies (Abcam [ab1791], at a final concentration of 13 μg/ml), and anti-H4 antibodies (Abcam [ab10158], at a final concentration of 13 μg/ml) were used for immunoprecipitation. The bead washes were performed for 15 min.

### ChIP-seq. (i) Library preparation.

ChIP DNA obtained from CENP-A^Cse4^–protein A (4 ng) was used to generate a sequencing library using NEBNext Ultra II DNA Library Prep kit for Illumina (catalog no. E7645S). In brief, the fragmented DNA was subjected to end repair followed by A-tailing and adapter ligation. The product DNA was enriched by PCR amplification using Illumina index adapter primers and purified using AMPure beads to remove unused primers. The library was quantitated using a Qubit DNA high-sensitivity quantitation assay, and the library quality was checked on a Bioanalyzer 2100 using an Agilent 7500 DNA kit.

### (ii) Data analysis.

ChIP-seq yielded 20,816,547 reads for the input, and 20,959,149 reads for IP. Based on the FastQC (v0.11.8) report, adaptor sequences and orphan reads were removed using Trim Galore! (v0.4.4) (http://www.bioinformatics.babraham.ac.uk/projects/). The output file was mapped onto the GenBank reference assembly for C. auris clade 1 (GCA_002759435.2) to obtain the sequence alignment map in SAM format. Conversion to BAM, sorting, and indexing was achieved using SAMtools (v1.9) (61). Identification and excision of duplicates were made using MarkDuplicates scripted by Picard tools (v1.119) (http://broadinstitute.github.io/picard/). The processed binary alignment map was used as input for MACS2 (v2.1.1) (62), along with the genome control reads (processed in the same way as the immunoprecipitation sample) to generate peaks. The peaks were then sorted based on the *P* value, the false discovery rate value, and the fold change. The peaks were visualized using Integrative Genomic Viewer (v2.4.1) (63). Enrichment peaks were curated (fold enrichment, ≥2.6), and the coordinates of the peaks obtained from MACS2 post-peak calling were used to extract sequences from the genome assemblies. The extracted sequences were scanned for repeats using SyMap (v4.2) (64), and the result was depicted as a dot plot.

### ChIP-qPCR analysis.

Real-time PCR was used to confirm CENP-A^Cse4^ enrichment and H3 depletion in the centromere sequences, using primers specific to centromeres and noncentromeric loci (listed in Table S3) and SensiFAST SYBR No ROX kit. Dilutions of 1:50 for input and 1:20 for the IP were used to determine CENP-A^Cse4^ enrichment. Dilutions of 1:50 for input and 1:5 for the IP were used to determine histone H3 and H4 occupancy. The program used the following sequence: 94°C for 2 min, 94°C for 30 s, appropriate *T_m_* for 30 s, and 72°C for 30 s for 30 cycles. The adjusted *C_T_* values (log_2_ of dilution factor subtracted from the *C_T_* value of the input or IP) were used to calculate the percentage input using the formula: 100 × 2^(*adjusted Ct of input − adjusted Ct of IP*)^. Three technical replicates were taken for the assay, and the standard error of the mean was calculated. The plots were generated using GraphPad Prism 8.

### Ortholog search and phylogenetic tree construction.

Available annotation files for S. cerevisiae (https://www.ncbi.nlm.nih.gov/assembly/GCF_000146045.2/), C. glabrata (https://www.ncbi.nlm.nih.gov/assembly/GCF_000002545.3/), C. albicans (https://www.ncbi.nlm.nih.gov/assembly/GCF_000182965.3/), C. tropicalis (https://www.ncbi.nlm.nih.gov/assembly/GCF_000006335.3/), C. dubliniensis (https://www.ncbi.nlm.nih.gov/assembly/GCF_000026945.1/), C. parapsilosis (https://www.ncbi.nlm.nih.gov/assembly/GCF_000182765.1/), *D. hansenii* (https://www.ncbi.nlm.nih.gov/assembly/GCF_000006445.2/), *S. stipitis* (https://www.ncbi.nlm.nih.gov/assembly/GCF_000209165.1/), C. neoformans (https://www.ncbi.nlm.nih.gov/assembly/GCF_000091045.1/), C. auris clade 1 (https://www.ncbi.nlm.nih.gov/assembly/GCA_002759435.2/), C. auris clade 2 (https://www.ncbi.nlm.nih.gov/assembly/GCA_003013715.2/), C. auris clade 4 (https://www.ncbi.nlm.nih.gov/assembly/GCA_008275145.1/), *C. duobushaemulonii* (https://www.ncbi.nlm.nih.gov/assembly/GCF_002926085.2/), *C. haemulonii* (https://www.ncbi.nlm.nih.gov/assembly/GCF_002926055.2/), *C. pseudohaemulonii* (https://www.ncbi.nlm.nih.gov/assembly/GCF_003013735.1/), C. lusitaniae (https://www.ncbi.nlm.nih.gov/assembly/GCF_000003835.1/), and *C. intermedia* (https://www.ncbi.nlm.nih.gov/assembly/GCA_900106115.1/) were downloaded from GenBank. Transcription and proteome data of C. lusitaniae were used to annotate the *C. fructus* (https://www.ncbi.nlm.nih.gov/assembly/GCA_003707795.1/) genome. C. auris clade 3 (https://www.ncbi.nlm.nih.gov/assembly/GCA_005234155.1/), *C. heveicola* (https://www.ncbi.nlm.nih.gov/assembly/GCA_003708405.1/), *C. oregonensis* (https://www.ncbi.nlm.nih.gov/assembly/GCA_003707785.2/), and *C. blattae* (https://www.ncbi.nlm.nih.gov/assembly/GCA_003706955.2/) genome assemblies were annotated using transcriptome and proteome data of C. auris clade 2, using MAKER (v2.31.10) (65). For all given species, clusters of orthologous proteins were identified using OrthoMCL (v2.0.9) (66). The single-copy orthologs present in all the species were identified and aligned using Clustal Omega (v1.2.4) (67). All the alignments were concatenated for each species, including the gaps. The gaps and corresponding sequences in all other species were removed. MrBayes (v2.3.5) (68) was used for tree construction, which was visualized using FigTree (v1.4.4) (http://tree.bio.ed.ac.uk/software/figtree/). Orthologs for proteins involved in heterochromatin formation and RNAi was done using phmmer option in HMMER (EMBL-EBI) (69).

### *In silico* analyses. (i) Gene synteny.

Centromere prediction in a candidate species was made by aligning the respective genome assembly to the reference species assembly using Mauve (Geneious v11.1.4; Biomatters, Ltd.), and the conserved synteny blocks corresponding to the ORFs flanking centromeres in the reference assembly were identified. For confirming synteny conservation, candidate species-specific local genome databases were created using Geneious. BLAST analysis of five individual ORFs on either side of the centromeres in the reference species assembly was performed against the local genome database of the candidate species, using the protein sequences as queries. For genome-level comparison, coordinates of all the synteny blocks conserved between two species were obtained using SyMap (v4.2), and the circos plots were drawn using Circos (v0.69-8) (70). Scaffold-level and ORF-level synteny analyses identifying rearrangements were done using Easyfig (v2.2.2) (71).

### (ii) Centromere sequence features.

Python scripts were written to determine the GC% at the third position of codons. The percentages of G and C at the third position of codons (except the stop codons) were calculated, followed by calculating the average values in a sliding window of 10 ORFs. These values were plotted for each scaffold of the genome. Annotations that are not a multiple of three were not considered for the analysis. GC% was also calculated for the whole scaffolds with a window size of 5 kb and a sliding step of 1 kb. GC skew [(G − C)/(G + C)] and AT skew [(A – T)/(A + T)] were plotted for a region of 10 kb flanking the centromeres using a window size of 100 bp and a sliding step of 1 bp. The skew calculation was done in Julia (v1.2.0), and the plotting was done in R. The “geom_smooth” function with “gam” method in ggplot2 (72) was used to smoothen the curve.

To study trends in centromere sequence evolution in different clades of C. auris, protein sequences were extracted using agat_sp_extract_sequences.pl from the AGAT suite (https://github.com/NBISweden/AGAT), and orthologous genes found using rsd_search (73). Intergenic sequence that occurred between the same pair of orthologous genes in pairs were identified as orthologous intergenic sequence and aligned using FSA (74), which we previously found to have high specificity for true homology in aligning intergenic DNA sequence (75). In each of the pairwise alignments generated by FSA, sequence divergence was estimated as #mutations/#matches, where #matches is the number of positions where an aligned pair of nucleotides is reported; and #mutations is the number of match positions where the alignment is a mismatch. The means and sample standard deviations over all intergenic sequences were calculated and compared to the observed numbers in centromeres.

If available, the respective genome assembly annotation files were used to report the length of ORF-free regions. Otherwise, all predicted ORFs larger than 600 bp were considered as coding sequences. Motif search was done using MEME in the MEME Suite (76).

### (iii) Gene expression.

For determining the transcriptional status of centromeres, the raw sequencing reads (SRR6900290, SRR6900291, SRR6900292, and SRR6900293) (30) were aligned to the reference genome of clade 1 (GenBank assembly GCA_002759435.2) using HISAT2 (v2.1.0) (77). The aligned reads were then graphically visualized in the IGV to analyze gene expression levels at/around the centromeres on different chromosomes. For studying the transcriptional status of ORFs overlapping with or flanking the centromeres, the abundance of annotated transcripts was quantified using pseudo alignment program kallisto (v0.46.1) (78). The expression of genes around/overlapping the centromere in TPM (transcripts per million) were compared to the global gene expression level.

### Data availability.

ChIP-seq data have been deposited in NCBI under BioProject PRJNA612018.
