# Supplementary figures and images for: Functional and Comparative Analysis of Centromeres Reveals Clade-Specific Genome Rearrangements in Candida auris and a Chromosome Number Change in Related Species
Source: mBio. 2021 May 11;12(3):e00905-21. doi: 10.1128/mBio.00905-21 (PMC8262905; doi:10.1128/mBio.00905-21)

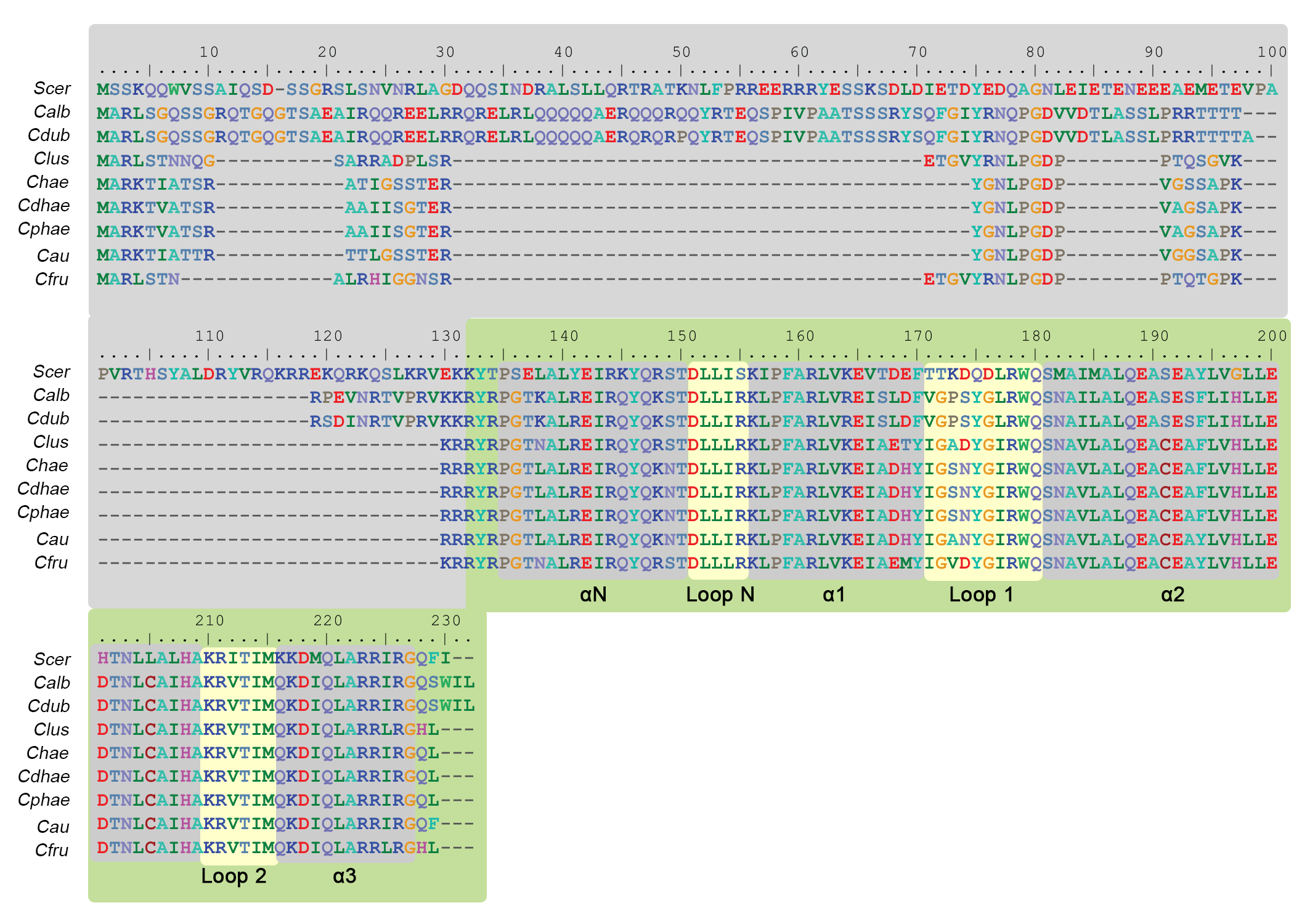

Supplement: FIG S1 [file mbio.00905-21-sf001.tif]

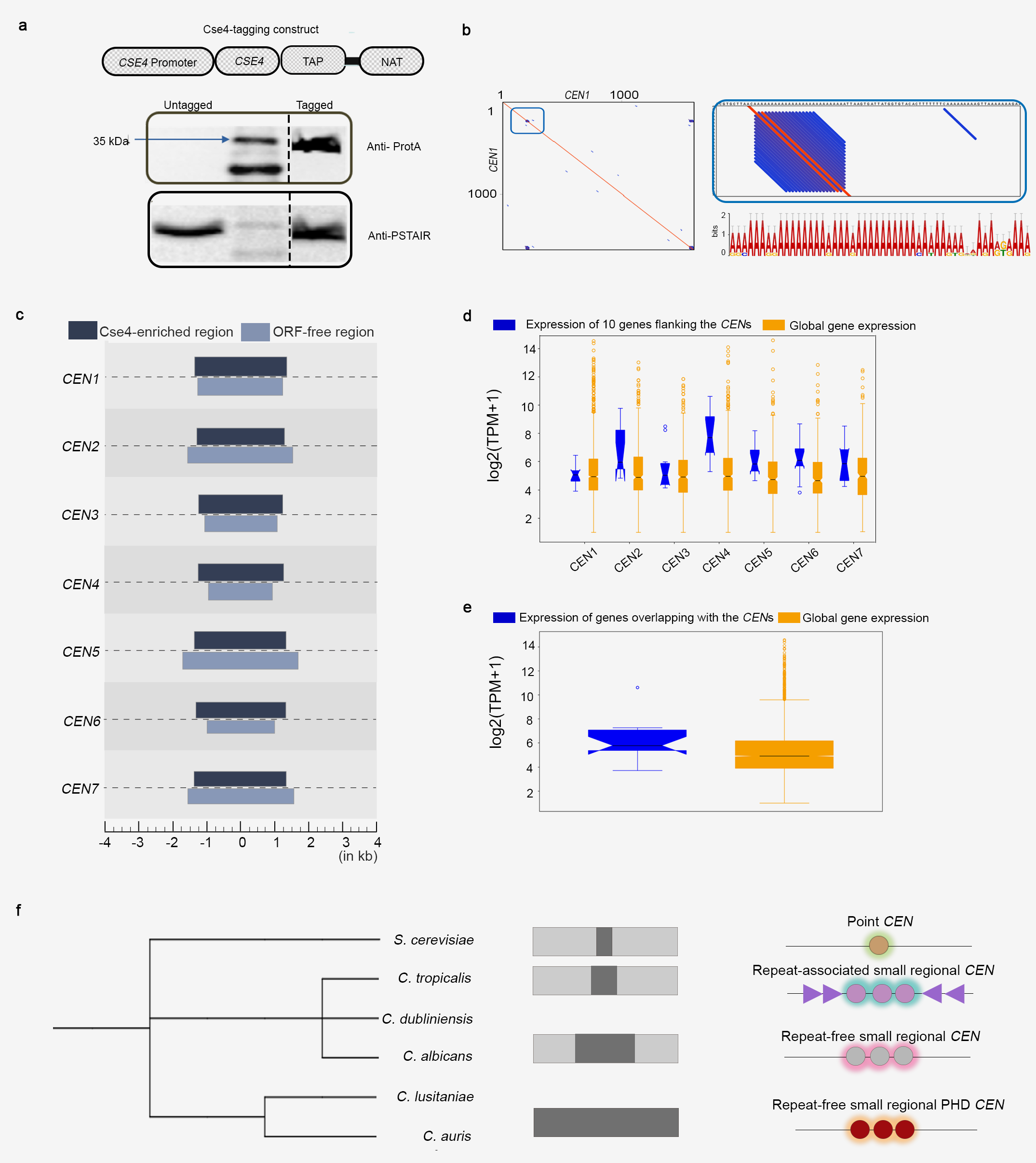

Supplement: FIG S2 [file mbio.00905-21-sf002.tif]

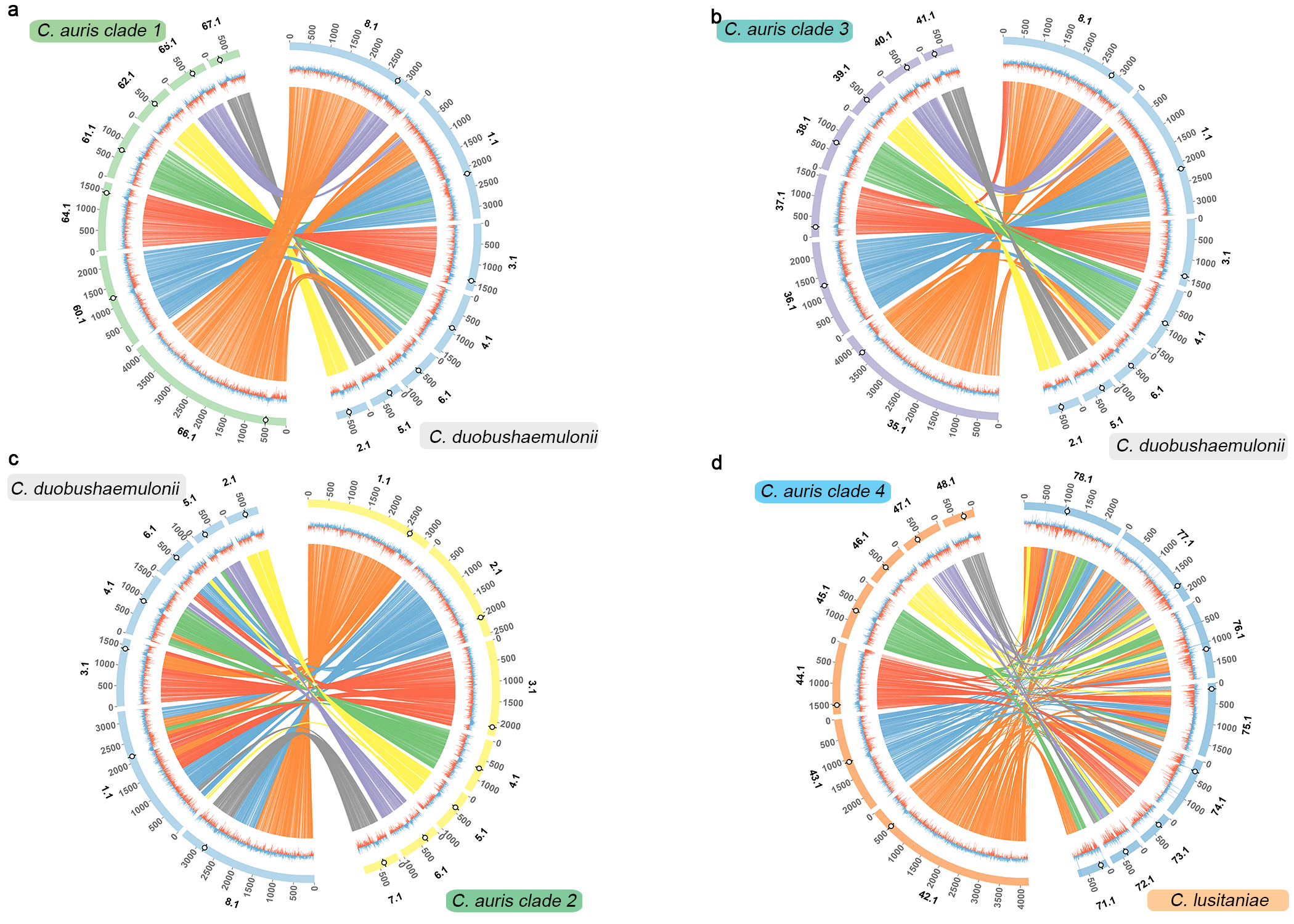

Supplement: FIG S3 [file mbio.00905-21-sf003.tif]

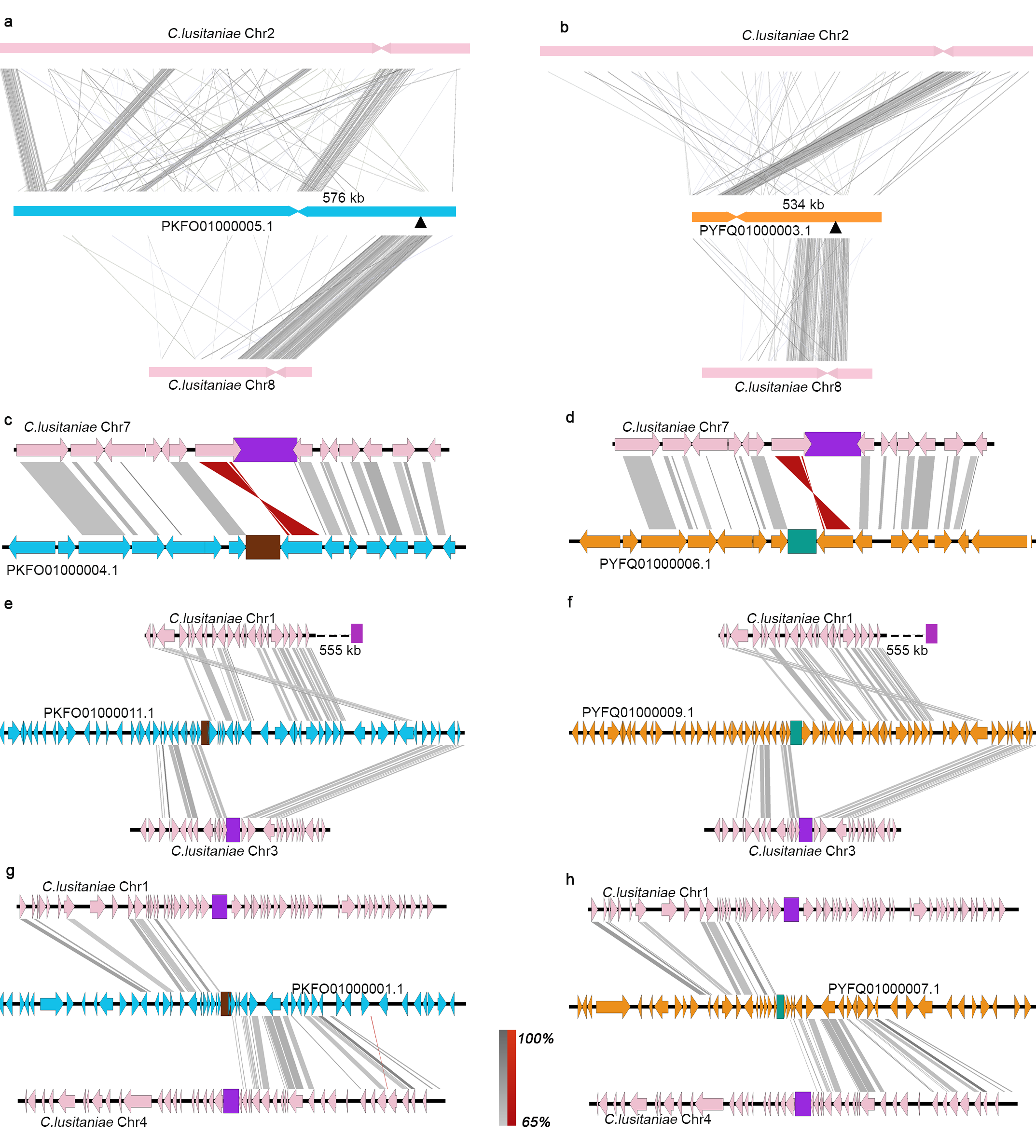

Supplement: FIG S4 [file mbio.00905-21-sf004.tif]

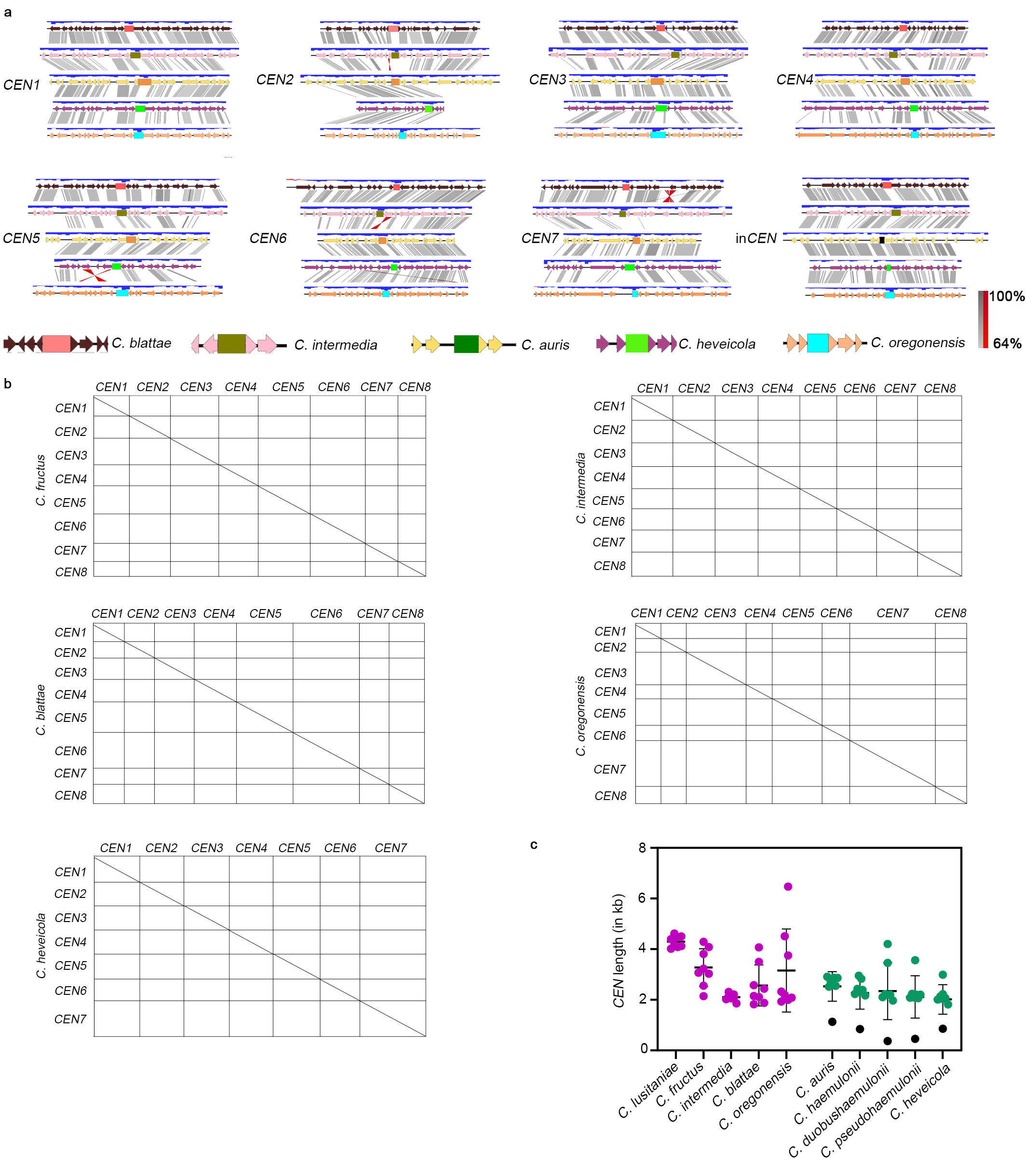

Supplement: FIG S5 [file mbio.00905-21-sf005.tif]
