# Supplementary material for: Functional and Comparative Analysis of Centromeres Reveals Clade-Specific Genome Rearrangements in Candida auris and a Chromosome Number Change in Related Species
Source: mBio. 2021 May 11;12(3):e00905-21. doi: 10.1128/mBio.00905-21 (PMC8262905; doi:10.1128/mBio.00905-21)
Supplement: TABLE S2 [file mbio.00905-21-st002.docx]

**Table S2: Oligonucleotides used in this study**

| Primer | Primer sequence (5’-3’) | Purpose |
| --- | --- | --- |
| D1-D2 seq FP | GCATATCAATAAGCGGAGGAAAAG | D1-D2 sequencing |
| D1-D2 seq RP | GGTCCGTGTTTCAAGACGG |  |
| TUB2 fp | AGATTACTCACTCCTTGGGT | MLST analysis |
| TUB2 rp | AGACAAGGTCGCGTTATATG |  |
| EFB1 fp | CTCAATGACCAAGTTGATCTG |  |
| EFB1 rp | AACTTGGATGAGTTGTTGGC |  |
| RPB1 fp | AAGAGCAAAATGGTCTGTGA |  |
| RPB1 rp | GCTCGTTTTCTTCAAACTGT |  |
| Au CEN1 fp | CCGATACAGTACATCGTTGA | ChIP-qPCR  *CEN* primers |
| Au CEN1 rp | TGATCAGTGGCGTTCAATAA |  |
| Au CEN1 fp | TGATAGACTTTTCTGGTGGGT | ChIP-qPCR  *CEN* primers  (clade 4) |
| Au CEN1 rp | TTCTACTTTATTACATGGCTTTGC |  |
| Au CEN2 fp | CCATTCGTTGCTTCAATCAT | ChIP-qPCR  *CEN* primers |
| Au CEN2 rp | TCGTCTATCGCTTCATACAC |  |
| Au CEN3 fp | CACGGTAATGGATTGACACTA | ChIP-qPCR  *CEN* primers |
| Au CEN3 rp | GTCAGGATTTCAGTGATGCT |  |
| Au CEN4 fp | TGATAGACTTTTCTGGTGGGT | ChIP-qPCR  *CEN* primers |
| Au CEN4 rp | TTCTACTTTATTACATGGCTTTGC |  |
| Au CEN5 fp | AACAGGCACACAGTCAGATG | ChIP-qPCR  *CEN* primers |
| Au CEN5 rp | CTGCGTAAGCTGAAAAACCG |  |
| Au CEN6 fp | ACACATTTACTTTTTGACGGG | ChIP-qPCR  *CEN* primers |
| Au CEN6 rp | GGGTAAGAATCGTGAGAGAA |  |
| Au CEN7 fp | GTAACCCATGTGGAGCACAA | ChIP-qPCR  *CEN* primers |
| Au CEN7 rp | CGGCCATGCATCAATCCTAT |  |
| Au CEN7 fp | TTCCATTCTCCAAGGATAGG | ChIP-qPCR  *CEN* primers  (clade 4) |
| Au CEN7 rp | TATCTAAATGCAATCGTGGG |  |
| Au 4532 fp | TATCTCTTGAGCTGGATGGT | ChIP-qPCR  Control region |
| Au 4532 rp | AACTTCCTGCTGGACAAAAT |  |
| Duo CEN1 fp | GGTCACTTATACGAACACCA | ChIP-qPCR  *CEN* primers |
| Duo CEN1 rp | TTACACGAGCTGCTATTACC |  |
| Duo CEN2 fp | CACAATCCTGTGATCTAGCA | ChIP-qPCR  *CEN* primers |
| Duo CEN2 rp | AATACGAAGCACTTCAACCT |  |
| Duo CEN3 fp | TTAACTGGTAATAGGGCACG | ChIP-qPCR  *CEN* primers |
| Duo CEN3 rp | AAAAAGTACGAAAACCGAGC |  |
| Duo CEN4 fp | ATGACCTAGGGACATCTTCT | ChIP-qPCR  *CEN* primers |
| Duo CEN4 rp | ATTTTGGAGACGACCATTTC |  |
| Duo 4532 fp | TAGATGTCAGGTGGTCAGAA | ChIP-qPCR  Control region |
| Duo 4532 rp | CGTTTTCATCAACACGCATA |  |
| Hae CEN2 fp | TTCTAAACAAACGTACCCGA | ChIP-qPCR  *CEN* primers |
| Hae CEN2 rp | AGCTAGGAGATGAAATCCGA |  |
| Hae CEN3 fp | ATGTAACGTCAATGGTGACA | ChIP-qPCR  *CEN* primers |
| Hae CEN3 rp | GCTCACAGTTTGTCTTAGGT |  |
| Hae CEN4 fp | TCTTAGTATGCCGTTGTAGC | ChIP-qPCR  *CEN* primers |
| Hae CEN4 rp | CGCCCAATCAAACACTAAC |  |
| Hae CEN6 fp | GCCTTTATGTCATGTTGTGC | ChIP-qPCR  *CEN* primers |
| Hae CEN6 rp | AACTATGTAGGCTGTTGCAT |  |
| Hae CEN7 fp | AGAGAAGTAGGTAGCTTGGA | ChIP-qPCR  *CEN* primers |
| Hae CEN7 rp | ATATGACCACATCAATCGGG |  |
| Hae 4532 fp | CCAGGAAGGTATTGAGGCTA | ChIP-qPCR  Control region |
| Hae 4532 rp | CCAACGTAGTCTACTCTTCTG |  |
| Phae CEN2 fp | CCTACTTCTGGCGTTCATAA | ChIP-qPCR  *CEN* primers |
| Phae CEN2 rp | AAAGCCTGCATTATTTGTCC |  |
| Phae CEN4 fp | CCATTCCCCTCCTTATTTGT | ChIP-qPCR  *CEN* primers |
| Phae CEN4 rp | GGAACTACAACCACCTGAAT |  |
| Phae CEN6 fp | ATTAGAAGCCTTGTTCGAGAT | ChIP-qPCR  *CEN* primers |
| Phae CEN6 rp | AACTAGAGACACCAACAGAG |  |
| Phae CEN7 fp | TTCTTAACAAGTGGTCGAGG | ChIP-qPCR  *CEN* primers |
| Phae CEN7 rp | AATCCGATCCACAACTTCAC |  |
| Phae 4532 fp | AACCACTACACATGGTCTTC | ChIP-qPCR  Control region |
| Phae 4532 fp | TCTTAGAAGCCTCCAAGTCT |  |
| CSE4-TAP-US-FP | AGCTGGTACCATGAGGATGGTGATTGAATCATC | Strain construction |
| CSE4-TAP-US-RP | agctACTAGTGAATTGACCTCGTATCCTTCG |  |
| CSE4-TAP-DS-FP | AGCTACTAGTATGAGAACTTTAGAGAATTGTGTC |  |
| CSE4-TAP-DS-RP | AGCTGCGGCCGCTCTGAGTCATAATATTCCGAGTC |  |
| CSE4-TAP- CN | GCTCAACAATACCGCAAATGTTATC |  |
