# Supplementary material for: Functional and Comparative Analysis of Centromeres Reveals Clade-Specific Genome Rearrangements in Candida auris and a Chromosome Number Change in Related Species
Source: mBio. 2021 May 11;12(3):e00905-21. doi: 10.1128/mBio.00905-21 (PMC8262905; doi:10.1128/mBio.00905-21)
Supplement: TABLE S3 [file mbio.00905-21-st003.docx]

**Table S3: Centromere sequence divergence in different geographical clades**

| Sequence divergence | | | | | | | | | | | | |
| --- | --- | --- | --- | --- | --- | --- | --- | --- | --- | --- | --- | --- |
|  | **CENs (Z score)** | | | | **Inactive CENs (Z score)** | | | | **Intergenic (standard deviation)** | | | |
| Clade | 1 | 2 | 3 | 4 | 1 | 2 | 3 | 4 | 1 | 2 | 3 | 4 |
| 1 | 0 | 0.025 (0.273) | 0.092 (1.863) | 0.049 (0.583) | 0 | 0.011  (-0.131) | 0.082 (1.615) | 0.014  (-0.575) | 0 | 0.015 (0.034) | 0.013 (0.042) | 0.032 (0.030) |
| 2 | 0.025 (0.273) | 0 | 0.078 (5.119) | 0.051 (0.218) | 0.011  (-0.131) | 0 | 0.078 (5.125) | 0.020  (-0.497) | 0.015 (0.034) | 0 | 0.009 (0.013) | 0.042 (0.044) |
| 3 | 0.092 (1.863) | 0.078 (5.119) | 0 | 0.121 (5.106) | 0.082 (1.615) | 0.078 (5.125) | 0 | 0.089 (3.395) | 0.013 (0.042) | 0.009 (0.013) | 0 | 0.025 (0.019) |
| 4 | 0.049 (0.583) | 0.051 (0.218) | 0.121 (5.106) | 0 | 0.014  (-0.575) | 0.020  (-0.497) | 0.089 (3.395) | 0 | 0.032 (0.030) | 0.042 (0.044) | 0.025 (0.019) | 0 |

Standard deviation for sequence divergence at intergenic region is shown, and the values at the centromeres are shown as Z-scores (difference from mean in units of standard deviation).
