# Supplementary material for: Functional and Comparative Analysis of Centromeres Reveals Clade-Specific Genome Rearrangements in Candida auris and a Chromosome Number Change in Related Species
Source: mBio. 2021 May 11;12(3):e00905-21. doi: 10.1128/mBio.00905-21 (PMC8262905; doi:10.1128/mBio.00905-21)
Supplement: TABLE S4 [file mbio.00905-21-st004.docx]

**Table S4: Centromeres in related species – scaffold map**

| *C. lusitaniae CEN* | Scaffold # in *C. fructus* assembly  (coordinates) | *C. auris CEN* | Scaffold # in *C. intermedia* assembly  (coordinates) | Scaffold # in *C. blattae* assembly  (coordinates) | Scaffold # in *C. heveicola* assembly  (coordinates) | Scaffold # in *C. oregonensis* assembly  (coordinates) |
| --- | --- | --- | --- | --- | --- | --- |
| *CEN1* | PPLK01000041.1  (24682-27714) | *CEN1* | LT635762.1  (97293-99600) | PPMS02000002.1  (509108-511216) | PPOB01000009.1  (395236-397457) | PPLJ02000002.1  (391512-393700) |
| *CEN2* | PPLK01000014.1  (61157-64387) | *CEN2* | LT635756.1  (2116097-2118190) | PPMS02000018.1  (27373-29949) | PPOB01000012.1  (447091-449119) | PPLJ02000010.1  (186066-187999) |
| *CEN3* | PPLK01000020.1  (124314-128121) | *CEN3* | LT635761.1  (421692-423715) | PPMS02000004.1  (603681-607751) | PPOB01000002.1  (233988-236058) | PPLJ02000003.1  (203021-207534) |
| *CEN4* | PPLK01000045.1  (70020-73092) | *CEN4* | LT635756.1  (750835-752965) | PPMS02000017.1  (90553-92428) | PPOB01000010.1  (283258-285269) | PPLJ02000005.1  (151694-153692) |
| *CEN5* | PPLK01000004.1  (293788-297868) | *CEN5* | LT635758.1  (677659-679836) | PPMS02000014.1  (222093-224527) | PPOB01000015.1  (267746-269874) | PPLJ02000008.1  (277804-281557) |
| *CEN6* | PPLK01000003.1  (198330-202619) | *CEN6* | LT635757.1  (953334-955390) | PPMS02000019.1  (35853-37676) | PPOB01000004.1  (375189-377003) | PPLJ02000001.1  (1139147-1141232) |
| *CEN7* | PPLK01000010.1  (54115-56673) | *CEN7* | LT635759.1  (597158-599012) | PPMS02000001.1  (505461-508960) | PPOB01000006.1  (552389-555379) | PPLJ02000007.1  (451609-458079) |
| *CEN8* | PPLK01000011.1  (43113-45253) | in*CEN* | LT635760.1  (826270-828478***) | PPMS02000005.1  (142874-145022*) |  | PPLJ02000013.1  (162814-165140*) |

*: No sequence loss
