# Supplementary material for: Functional and Comparative Analysis of Centromeres Reveals Clade-Specific Genome Rearrangements in Candida auris and a Chromosome Number Change in Related Species
Source: mBio. 2021 May 11;12(3):e00905-21. doi: 10.1128/mBio.00905-21 (PMC8262905; doi:10.1128/mBio.00905-21)
Supplement: TABLE S5 [file mbio.00905-21-st005.docx]

**Table S5: Distribution of *C. lusitaniae* *CEN8*-containing synteny block in *C. auris* clades and related species**

| Synteny block in *C. lusitaniae*  Scaffold (start-end) | Size (in bp) | Synteny block in | Scaffold (start-end) | Size (in bp) |
| --- | --- | --- | --- | --- |
| CH408083.1  (89697-359662) | 269965 | *C. auris* clade 1 | PEKT02000010.1 (1066463-1295990) | 229527 |
| CH408083.1  (89707-217179) | 127472 | *C. auris* clade 2 | CP043536.1 (127217-235434) | 108217 |
| CH408083.1  (243462-359663) | 116201 | *C. auris* clade 2 | CP043534.1 (1076434-1178122) | 101688 |
| CH408083.1  (89707-359645) | 269938 | *C. auris* clade 3 | CM016500.1 (1065258-1294427) | 229169 |
| CH408083.1  (89707-359663) | 269956 | *C. auris* clade 4 | CP043445.1  (134688-363058) | 228370 |
| CH408083.1  (89745-359649) | 269904 | *C. haemulonii* | PKFO01000005.1  (1736412-1973270) | 236858 |
| CH408083.1  (89640-359720) | 270080 | *C. duobushaemulonii* | PKFP01000004.1  (1235993-1464114) | 228121 |
| CH408083.1  (89719-356554) | 266835 | *C. pseudohaemulonii* | PYFQ01000003.1  (485191-714677) | 229486 |
