# Supplementary material for: Functional and Comparative Analysis of Centromeres Reveals Clade-Specific Genome Rearrangements in Candida auris and a Chromosome Number Change in Related Species
Source: mBio. 2021 May 11;12(3):e00905-21. doi: 10.1128/mBio.00905-21 (PMC8262905; doi:10.1128/mBio.00905-21)
Supplement: TABLE S1 [file mbio.00905-21-st001.docx]

**Table S1: Strains used in this study**

| Strain | Species | Description | Source |
| --- | --- | --- | --- |
| Cau46R | *C. auris* | Clade 1 clinical isolate | NCCPF*, PGIMER, India |
| CBS1091131T | *C. auris* | Clade 2 strain | CBS |
| AR-0383 | *C. auris* | Clade 3 strain | Rutgers University |
| AR-0385 | *C. auris* | Clade 4 strain | Rutgers University |
| CauI46 | *C. auris* | *CSE4-TAP:: NAT* | This study |
| NCCPF470162 | *C. haemulonii* | Clinical isolate | NCCPF, PGIMER, India |
| NCCPF470163 | *C. pseudohaemulonii* | Clinical isolate | NCCPF, PGIMER, India |
| NCCPF470164 | *C. duobushaemulonii* | Clinical isolate | NCCPF, PGIMER, India |

*NCCPF- National Culture Collection of Pathogenic Fungi
